# Associations between the implementation of telework strategies and job performance: Moderating influences of boundary management preferences and telework experience

**DOI:** 10.3389/fpsyg.2023.1099138

**Published:** 2023-02-16

**Authors:** Tobias M. Härtel, Dominik Hüttemann, Julia Müller

**Affiliations:** School of Business Administration and Economics, Chair of Corporate Management, Osnabrück University, Osnabrück, Germany

**Keywords:** telework, telework strategies, job performance, boundary theory, boundary management preferences, telework experience, person-environment fit

## Abstract

Boosted by the COVID-19 pandemic, more than ever, an organization’s success depends on its teleworkers’ performance. However, little attention has been paid to the individual strategies implemented by teleworkers to achieve goals such as drawing boundaries between work- and private-life, working task-oriented and productively, and keeping social contact. We collected quantitative survey data of 548 teleworkers indicating their implementation of 85 telework strategies derived from scientific literature and popular media (e.g., working in a separate room, wearing work clothes at home), self-reported job performance, boundary management preferences, and telework experience. We identified (a) the implementation of telework strategies, (b) associations with job performance, (c) divergences between the implementation and the performance association, and (d) moderating influences of boundary management preferences and telework experience. The results suggest that the most implemented telework strategies tend to be the ones most positively associated with job performance. These telework strategies serve goals related to working task-oriented and productively by adopting a conducive work attitude as well as keeping social contact by using modern communication technology rather than goals related to drawing boundaries between work- and private-life. The findings underscore the benefits of expanding a narrow focus on telework strategies stemming from boundary theory to unravel telework strategies’ puzzling impacts on (tele-) work outcomes. Also, taking a person-environment fit perspective appeared to be a promising approach to tailor evidence-based best practice telework strategies to teleworkers’ individual preferences and needs (boundary management preferences and telework experience).

## 1. Introduction

Teleworking has become a popular work mode (Allen et al., 2015) and its prevalence has recently been further boosted by the COVID-19 pandemic (Kramer and Kramer, 2020; Kniffin et al., 2021; Milasi et al., 2021; Rudolph et al., 2021). Thus, more than ever before, an organization’s success depends on its teleworkers’ performance and this trend is likely to continue due to demographic workforce changes, widespread distribution of information communication technology, as well as sustainability and work-life balance considerations (Athanasiadou and Theriou, 2021).

Some exploratory qualitative research (e.g., Fonner and Stache, 2012; Basile and Beauregard, 2016) has identified telework strategies, that is, individual strategies teleworkers implement when organizing their telework, that might impact work outcomes such as job performance. Also, the popular media is full of telework strategies (often referred to as “tips and tricks for working from home”) that are suggested to enhance job performance. Examples of such telework strategies are using a separate room for teleworking or wearing work clothes at home. With this study, we respond to multiple calls for research on the differential impacts of the implementation of telework strategies on (tele-) work outcomes and potentially moderating factors (Allen et al., 2021; Rudolph et al., 2021; see also Binnewies et al., 2020). More specifically, we address blank spots of previous research on telework strategies by providing empirical evidence on (a) how much telework strategies are implemented, (b) how the implementation of telework strategies is associated with job performance, (c) divergences between the telework strategies’ implementation and association with job performance, and (d) how the association between the implementation of telework strategies and job performance is moderated by teleworker characteristics such as boundary management preferences and telework experience.

Overall, this study advances the young literature on telework strategies by demonstrating that extending a narrow focus on telework strategies stemming from boundary theory (Nippert-Eng, 1996; Ashforth et al., 2000) with telework strategies focusing on goals such as working productively (e.g., Greer and Payne, 2014) by adopting a conducive work attitude and keeping social contact (e.g., Kowalski and Swanson, 2005) by using modern communication technology might be a fruitful avenue for research illuminating impacts of telework strategies on work outcomes. Also, taking a person-environment fit perspective (Kristof, 1996; Edwards, 2008), particularly boundary congruence/fit (Kreiner, 2006; Ammons, 2013), appeared to be a promising approach to identifying evidence-based best practice telework strategies taking individual teleworker characteristics (boundary management preferences and telework experience) into account.

### 1.1. Implementation of telework strategies

*Telework* is a work practice enabling employees (*teleworkers*) to conduct all or a share of their work away from their on-site workplace, typically from home (Allen et al., 2015). Whereas numerous studies examined the impacts of teleworking (Gajendran and Harrison, 2007), such as reduced work-family conflict (e.g., Raghuram and Wiesenfeld, 2004; Golden et al., 2006; Allen et al., 2013), enhanced job performance (e.g., Bailey and Kurland, 2002; Bloom et al., 2015; Gajendran et al., 2015), and professional isolation (e.g., Baruch and Nicholson, 1997; Gainey and Kelley, 1999; Kurland and Cooper, 2002), little attention has been paid to the individual strategies teleworkers implement (*telework strategies*) when organizing their telework. These telework strategies may serve different goals such as drawing boundaries between work- and private-life (e.g., Fonner and Stache, 2012; Basile and Beauregard, 2016; Golden, 2021; see also Allen et al., 2021, calling for research), working task-oriented and productively (e.g., Greer and Payne, 2014; Troll et al., 2022), and keeping social contact (e.g., Ilozor et al., 2001; Turetken et al., 2011).

Most research on telework strategies stems from *boundary theory* (Nippert-Eng, 1996; Ashforth et al., 2000) proposing that individuals follow idiosyncratic approaches (*boundary management strategies*) to establish or dismantle boundaries in order to organize transitions between their work- and private-life. In an interview-based landmark study, Kreiner et al. (2009) distinguished four categories of boundary management strategies implemented by priests: Physical (manipulating physical space/items, e.g., setting up a separate workstation), temporal (manipulating time, e.g., setting work/non-work times), behavioral (inter alia establishing technological routines, e.g., not taking work-related calls after hours), and communicative (setting expectations and making arrangements, e.g., confronting boundary violators) boundary management strategies. Basile and Beauregard (2016) applied boundary management strategies to the telework context, in which boundaries between work- and private-life are particularly prone to blur. They found qualitative evidence for the implementation of physical (e.g., mimicking the physical boundary of an on-site office at home), temporal (e.g., establishing set times to finish the workday at home), behavioral (e.g., recreating technological routines of stationary work to ending up the workday at home), and communicative (e.g., making arrangements with household members facilitating undisturbed work at home) telework strategies. Other qualitative studies found similar telework strategies to be implemented (Tietze, 2002; Tietze and Musson, 2003; Myrie and Daly, 2009; Mustafa, 2010; Nansen et al., 2010; Fonner and Stache, 2012; Mustafa and Gold, 2013; Allen et al., 2021). First quantitative studies provide initial evidence on boundary related (telework) strategies’ global (Kossek et al., 2006; Wepfer et al., 2018) and differentiated level of implementation (differentiating between the aforementioned categories; Binnewies et al., 2020; Park et al., 2020; Haun et al., 2022) along with impacts on outcomes such as family-to-work conflict, recovery experiences, and well-being.

Whereas most studies embedded telework strategies in the framework of boundary theory, Greer and Payne (2014) identified complementing telework strategies by asking 86 high-performing teleworkers to freely recall telework strategies facilitating task-oriented and productive telework. Keeping connected with colleagues, supervisors, and customers (e.g., being accessible via various communication channels), using modern technologies (e.g., using a technological setup at home close to the on-site setup), and showing a conducive work attitude (e.g., adopting a work-oriented mindset) were frequently mentioned. Taking a quantitative approach, Troll et al. (2022) recently found telework strategies related to self-control (Duckworth et al., 2014), in particular, altering somatic conditions (optimizing the physical state to work productively, e.g., sleeping sufficiently) and autonomous motivation (motivating oneself to start and endure work tasks), to be frequently implemented and associated with working productively among 106 teleworkers.

Furthermore, some research has found telework strategies related to keeping social contact (e.g., seeking social interaction; Kowalski and Swanson, 2005) to be associated with job satisfaction (Ilozor et al., 2001), knowledge sharing (Golden and Raghuram, 2010), and the reduction of social isolation (Mann et al., 2000). In a similar vein, building on media richness theory (MRT; Daft and Lengel, 1986), Turetken et al. (2011) examined the impacts of telework strategies related to communication media richness (i.e., the extent to which a medium approximates face-to-face communication).

Overall, mainly qualitative approaches were used to identify telework strategies stemming from different theoretical frameworks and pursuing different goals, particularly establishing boundaries between work- and private-life (Nippert-Eng, 1996; Tietze, 2002; Tietze and Musson, 2003; Kossek et al., 2006; Kreiner et al., 2009; Myrie and Daly, 2009; Nansen et al., 2010; Fonner and Stache, 2012; Basile and Beauregard, 2016; Wepfer et al., 2018; Binnewies et al., 2020; Park et al., 2020; Golden, 2021; Haun et al., 2022), but also working task-oriented and productively (Greer and Payne, 2014; Troll et al., 2022), and keeping social contact (Mann et al., 2000; Ilozor et al., 2001; Kowalski and Swanson, 2005; Golden and Raghuram, 2010; Turetken et al., 2011). Whereas qualitative approaches are suited to exploratively identify telework strategies, they come with limitations that can be targeted by quantitative approaches: First, participants might forget to mention implemented telework strategies. Second, the binary classification of (not) implementing a telework strategy does not display gradual differences. Both aspects impede the identification of the impacts of implementing telework strategies. Quantitative research, however, is scarce and has either been conducted outside the telework context (Wepfer et al., 2018; Binnewies et al., 2020), or has placed a narrow focus on a specific facet of telework strategies, namely, telework strategies related to boundary management (Haun et al., 2022), to self-control (Troll et al., 2022), or to keeping social contact (Mann et al., 2000; Ilozor et al., 2001; Kowalski and Swanson, 2005; Golden and Raghuram, 2010; Turetken et al., 2011). Also, previous research reported results solely on an aggregated level differentiating between broad telework strategy categories.

**Figure 1:**
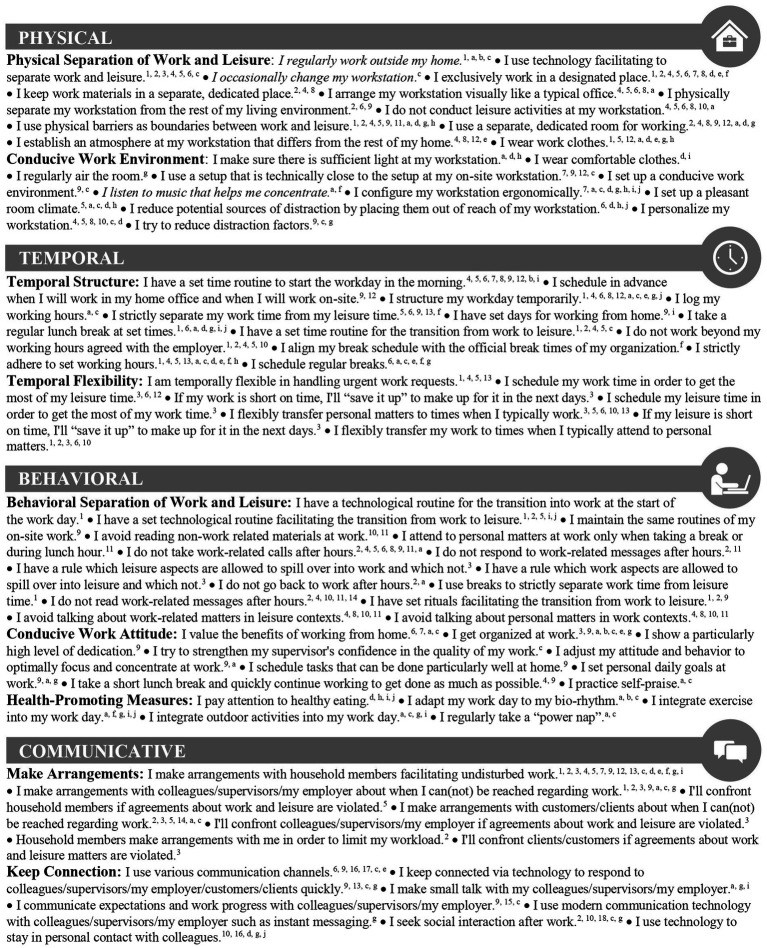
Overview of telework strategies. *Note.* Telework strategies in italics were recoded. Telework strategies extracted from scientific literature (popular media) were indicated with numbers (letters). ^1^Fonner and Stache (2012); ^2^Basile and Beauregard (2016); ^3^Kreiner et al. (2009); ^4^Mustafa and Gold (2013); ^5^Myrie and Daly (2009); ^6^Nansen et al. (2010); ^7^Kowalski and Swanson (2005); ^8^Mustafa (2010); ^9^Greer and Payne (2014); ^10^Wepfer et al. (2018); ^11^Kossek et al. (2006); ^12^Tietze (2002); ^13^Tietze and Musson (2003); ^14^Park et al. (2020); ^15^Ilozor et al. (2001); ^16^Golden and Raghuram (2010); ^17^Turetken et al. (2011); ^18^Mann et al. (2000). ^a^Mai (n.d.); ^b^Prophet (2017); ^c^Schulz (2020); ^d^Cobler (n.d.); ^e^Stross (n.d.); ^f^Westdeutsche Zeitung (2020); ^g^Flatley (2020); ^h^Vollmer (2018); ^i^Unger (2020); ^j^Rewe (n.d.).

With this study, we advance the young literature on telework strategies by (a) quantitatively examining a comprehensive set of 85 telework strategies stemming from different theoretical streams and pursuing different goals, and (b) thereby conducting analyses on both an aggregated category level to identify overarching patterns (Binnewies et al., 2020; Haun et al., 2022; Troll et al., 2022), and on an individual telework strategy level to draw highly resolved, zoomed-in inferences. To paint a comprehensive picture, we complement telework strategies derived from the scientific literature with telework strategies from popular media (see Figure 1). Online practical guides on telework strategies have flourished during the COVID-19 pandemic and many teleworkers have presumably been searching for advice.

### 1.2. Associations between telework strategies and job performance

There is initial evidence that the implementation of telework strategies is associated with outcomes such as well-being, recovery, satisfaction, knowledge sharing, and reduced isolation (Mann et al., 2000; Ilozor et al., 2001; Golden and Raghuram, 2010; Wepfer et al., 2018; Binnewies et al., 2020; Park et al., 2020; Haun et al., 2022). However, we know little about how the implementation of telework strategies is related to job performance. For instance, Binnewies et al. (2020) call for research on the consequences of boundary management strategies for job performance. Rudolph et al. (2021, p. 13) call for research on telework strategies and state that “it would be useful to have empirical information on the efficacy”. Allen et al. (2021, p. 81) conclude that “additional work is needed that provides guidance concerning the effectiveness of various strategies”.

Kossek et al. (2006) provide initial evidence that boundary related telework strategies’ global implementation might not be associated with job performance. Greer and Payne (2014) provide first hints on telework strategies freely-recalled by high performers (in particular keeping connected, using modern technology, showing a conducive work attitude) that might be positively associated with job performance. However, to reliably identify telework strategies associated with job performance, the inclusion of low performers is needed to (a) rule out that low performers use the same telework strategies as high performers, (b) identify telework strategies that might deteriorate job performance, and (c) make use of the full job performance range facilitating to detect significant associations by mitigating range restrictions. Troll et al. (2022) found self-control telework strategies related to autonomous motivation and somatic condition to be unique positive predictors of job performance. Turetken et al. (2011) found a positive association between telework strategies related to communication media richness and self-reported job performance.

In this study, we examine associations of a broad set of highly resolved telework strategies with job performance to paint a comprehensive, fine-grained picture. As we examine both the implementation of telework strategies and their association with job performance, this also offers the possibility to examine whether teleworkers have an intuitive understanding of telework strategies’ relation to job performance, that is, whether telework strategies that are more (less) associated with job performance are implemented more (less). In particular, telework strategies can then be identified that are “under (over) implemented”, that is, that are implemented less (more) frequently than they actually should be according to their high (low) association with job performance.

### 1.3. Moderating influences of boundary management preferences and telework experience

It is crucial to understand which telework strategies might be more or less strongly related to job performance for specific groups of employees sharing common characteristics (see also Binnewies et al., 2020, calling for research on moderators of boundary management strategies) to provide advice or training interventions tailored to employees’ individual preferences and needs (see also Kossek, 2016). Following *person-environment fit approaches* (P-E fit; Kristof, 1996; Edwards, 2008; see also Arthur et al., 2006), a (mis-) match between person and environment induces additional effects beyond the respective separate main effects. More specifically, the preferences/needs-supplies fit considers individual preferences and needs (here related to boundary management preferences and telework experience) and environmental supplies (here related to telework strategies) interacting to predict work outcomes (here job performance).

Boundary management preferences represent the degree to which employees prefer to separate (versus integrate) work- and private-life (Ashforth et al., 2000; Kreiner, 2006): Whereas employees leaning toward separation (“separators”) aim to draw rigorous boundaries, employees leaning toward integration (“integrators”) aim to remove boundaries. Individual boundary management preferences are crucial in telework contexts (Kossek et al., 2006; Allen et al., 2021; Kerman et al., 2022) because borders between work- and private-life are particularly prone to blur. Following P-E fit approaches, telework strategies congruent with individual boundary management preferences may be particularly beneficial to job performance because a fit between individual preferences and needs, and the environment is achieved. Thus, telework strategies aligned to separating work- and private-life (e.g., not working beyond agreed hours) might be more beneficial for separators, whereas telework strategies aligned to integrating work- and private-life (e.g., being flexible in handling work requests) might be more beneficial for integrators. This reasoning is supported by the *boundary congruence approach* (Kreiner, 2006) combining the P-E fit approach (Kristof, 1996; Edwards, 2008) with boundary theory (Nippert-Eng, 1996; Ashforth et al., 2000). Following the boundary congruence approach, a (mis-) fit (*boundary congruence* versus *boundary incongruence*) between individual boundary management preferences and environmental boundary influences such as workplace policies and conditions induces additional effects beyond the respective separate main effects (see Rothbard et al., 2005; Kreiner, 2006; Chen et al., 2009, for initial evidence on positive impacts of boundary congruence on outcomes such as reduced work-family conflict, higher job satisfaction, and commitment). In a similar vein, the *boundary fit approach* (Ammons, 2013) proposes that a (mis-) match (*boundary fit* versus *boundary misfit*) between individual boundary management preferences and boundary enactments (actual borders that individuals establish to separate work- and private-life) induces additional effects beyond the respective separate main effects (see Haun et al., 2022). In this study, we, for the first time, explore interaction effects related to boundary congruence/fit predicting job performance, whereby telework strategies might be either seen as environmental boundary influences or actual boundary enactments. During the COVID-19 pandemic, many employees were sent to the home office without choice (Kniffin et al., 2021) providing a unique opportunity to investigate moderating influences of boundary management preferences by mitigating self-selection effects: Typically, separators tend to prefer stationary on-site work over telework because telework is to some extent inherently incongruent with the preference to separate work- and private-life (Shockley and Allen, 2010).

The COVID-19 pandemic also provides the opportunity to examine moderating influences of the individual level of telework experience as many employees who have had little experience with telework migrated to the home office (Kramer and Kramer, 2020; Milasi et al., 2021). The wide range of experience levels allows us to examine moderating influences without self-selection biases and range restrictions. Following the P-E fit approach, teleworkers with low experience might have different needs than teleworkers with high experience so telework strategies might be differently beneficial for job performance. For instance, telework strategies providing structure (e.g., maintaining routines of the on-site stationary work) might be more beneficial for unexperienced teleworkers, whereas telework strategies demanding adaptivity (e.g., flexibly transferring work to times designated for personal matters) might be more beneficial for experienced teleworkers.

### 1.4. Present study

With this study, we shed light on the individual ways teleworkers organize their work processes to achieve different goals, in particular, drawing boundaries between work- and private-life, working task-oriented and productively, and keeping social contact. We aim to better understand (research question 1; RQ 1) the implementation of telework strategies, (RQ 2) associations with job performance, (RQ 3.1, 3.2) divergences between the implementation and association with job performance, and (RQ 4.1, 4.2) moderating influences of boundary management preferences and telework experience building on P-E fit theory. For this purpose, we collected survey data from 548 teleworkers assessing the implementation of 85 highly resolved telework strategies (see Figure 1), self-reported job performance, boundary management preferences, and telework experience. Due to the novelty and explorative nature of this research topic, and because this study was not preregistered, we do not formulate hypotheses but more open research questions:

- RQ 1: How much are telework strategies implemented?
- RQ 2: How is the individual implementation of telework strategies associated with job performance?
- RQ 3.1/3.2: How is the telework strategies’ average implementation associated with the telework strategies’ association with job performance?/How does the telework strategies’ relative average implementation diverge from their relative association with job performance?
- RQ 4.1/4.2: How is the association between the individual implementation of telework strategies and job performance moderated by boundary management preferences/telework experience?

## 2. Method

### 2.1. Sample

Our final sample consists of 548 German-speaking teleworkers^1^ (336 women) from various sectors (the most represented are 16.61% IT, telecommunication, media; 11.13% health and social services; 9.85% research and development) and professions (the most represented are 48.18% highly skilled and 29.38% skilled employees). We recruited participants via posting the online survey in online professional (LinkedIn, Xing) and social (Facebook) network groups without offering compensation. Participants on average spend most of their weekly working days teleworking (*M* = 3.85, *SD* = 1.33). The average age is 39.91 (*SD* = 11.63). Most participants (339, 71.72%) hold a bachelor’s degree or higher. Participants have extensive years of work experience (*M* = 15.52, *SD* = 11.92) and their weekly contractual working hours (*M* = 35.77, *SD* = 7.42) are similar to the weekly working hours of German full-time employees. Data collection took place between July and December 2020 and thus, started five months after the onset of the COVID-19 pandemic declared by the World Health Organization. This should ensure that teleworkers who had been teleworking for the first time due to the pandemic have had the opportunity to develop telework strategies (Lally et al., 2010). For an overview of the survey structure and assessed variables see the Codebook at https://osf.io/gqpdf/.

### 2.2. Measures

#### 2.2.1. Telework strategies

We originally extracted 157 telework strategies from scientific literature (Mann et al., 2000; Ilozor et al., 2001; Tietze, 2002; Tietze and Musson, 2003; Kowalski and Swanson, 2005; Kossek et al., 2006; Kreiner et al., 2009; Myrie and Daly, 2009; Golden and Raghuram, 2010; Mustafa, 2010; Nansen et al., 2010; Turetken et al., 2011; Fonner and Stache, 2012; Mustafa and Gold, 2013; Greer and Payne, 2014; Basile and Beauregard, 2016; Wepfer et al., 2018; Park et al., 2020)^2^ and added 179 telework strategies from popular media (Cobler, n.d.; Mai, n.d.; Rewe, n.d.; Stross, n.d.; Prophet, 2017; Vollmer, 2018; Flatley, 2020; Schulz, 2020; Westdeutsche Zeitung, 2020; Unger, 2020) to get a comprehensive selection of highly resolved telework strategies. We extracted telework strategies from popular media by reviewing the ten first listed websites of a Google search using the keyword “home office tips” (in German). After eliminating redundant telework strategies and exotic telework strategies from popular media (e.g., playing online casino to revive attention; Westdeutsche Zeitung, 2020), we finally included 85 distinct telework strategies in our online survey (see Figure 1). Whenever necessary, we translated the telework strategies into German and reformulated them into questionnaire items (e.g., the physical telework strategy “recreating the physical boundary of an office environment by designating areas for work activities” reported in Basile and Beauregard, 2016, p. 106, was transformed into the item “I physically separate my workstation from the rest of my living environment.”). Participants indicated the extent to which they implement telework strategies on a 6-point Likert scale ranging from 1 (*not at all*) to 6 (*completely*). Participants could also indicate not being aware of a particular telework strategy leading to missing values. However, the implementation of telework strategies was answered by most participants for each telework strategy (*M* = 534.49, *SD* = 23.25, *Min* = 420, *Max* = 548).

To enhance the comparability of our results with previous research and to identify overarching patterns on a higher aggregated level, we categorized the 85 telework strategies into physical, temporal, behavioral, and communicative superordinate categories (see Kreiner et al., 2009; Basile and Beauregard, 2016). Because telework strategies within the respective assigned superordinate categories were still heterogeneous, we simultaneously distinguished nine subordinate categories (e.g., “physical separation of work and leisure” and “conducive work environment” within the superordinate physical category; see Figure 1). Following guidelines for exploratory analyses (Miller, 1995) Cronbach’s alpha was acceptable for all superordinate (0.66 ≤ α ≤ 0.84) and subordinate categories (0.56 ≤ α ≤ 0.84), except for temporal flexibility (α = 0.56; see Table 1). Three telework strategies (“I regularly work outside my home.”, “I occasionally change my workstation.” within physical separation of work and leisure; “I listen to music that helps me concentrate.” within conducive work environment) needed to be recoded as they were negatively correlated with the respective subordinate categories’ overall score.

**Table 1:** Telework strategies’ implementation, multiple linear regression results of telework strategies and interaction effects with boundary management preferences and telework experience predicting job performance, and divergences between telework strategies’ implementation and association with job performance.

Telework strategy	*M*	β_T_	β_T x BMP_	β_T x TE_	Δ*M*, β_T_
Physical (α = 0.84)	3.95 [3.80, 4.10]	0.07 [−0.02, 0.16]	−0.01 [−0.10, 0.07]	−0.02 [−0.11, 0.07]	−0.22
Physical separation of work and leisure (α = 0.83)	3.81 [3.64, 3.97]	0.07 [−0.03, 0.16]	0.00 [−0.08, 0.09]	−0.03 [−0.12, 0.05]	−0.09
*I regularly work outside my home.*	5.39 [5.30, 5.49]	0.09 [−0.01, 0.19]	0.08 [−0.01, 0.17]	**−0.12** [−0.22, −0.02]	−1.75
I use technology facilitating to separate work and leisure.	4.65 [4.51, 4.80]	**0.11** [0.01, 0.20]	0.02 [−0.07, 0.11]	0.02 [−0.07, 0.10]	−0.65
*I occasionally change my workstation.*	4.57 [4.43, 4.71]	**0.10** [0.01, 0.19]	0.05 [−0.04, 0.13]	0.00 [−0.09, 0.08]	−0.67
I exclusively work in a designated place.	4.51 [4.37, 4.64]	**0.14** [0.05, 0.23]	0.01 [−0.08, 0.09]	0.01 [−0.07, 0.09]	−0.07
I keep work materials in a separate, dedicated place.	4.46 [4.32, 4.60]	0.07 [−0.02, 0.17]	0.00 [−0.09, 0.08]	−0.06 [−0.15, 0.02]	−0.79
I arrange my workstation visually like a typical office.	3.68 [3.52, 3.83]	0.06 [−0.03, 0.15]	−0.03 [−0.11, 0.05]	**−0.09** [−0.18, 0.00]	0.02
I physically separate my workstation from the rest of my living environment.	3.57 [3.40, 3.74]	0.03 [−0.07, 0.12]	−0.02 [−0.11, 0.06]	0.03 [−0.06, 0.12]	−0.24
I do not conduct leisure activities at my workstation.	3.39 [3.24, 3.53]	**0.12** [0.02, 0.21]	−0.04 [−0.13, 0.04]	−0.04 [−0.12, 0.04]	0.95
I use physical barriers as boundaries between work and leisure.	3.27 [3.10, 3.44]	0.00 [−0.10, 0.10]	−0.03 [−0.11, 0.06]	−0.01 [−0.10, 0.08]	−0.19
I use a separate, dedicated room for working.	3.21 [3.03, 3.40]	0.01 [−0.09, 0.10]	−0.03 [−0.12, 0.06]	−0.01 [−0.09, 0.08]	−0.05
I establish an atmosphere at my workstation that differs from the rest of my home.	2.58 [2.44, 2.73]	0.05 [−0.04, 0.15]	0.00 [−0.09, 0.09]	−0.05 [−0.14, 0.03]	1.23
I wear work clothes.	2.37 [2.23, 2.50]	0.02 [−0.07, 0.11]	0.06 [−0.03, 0.15]	−0.06 [−0.15, 0.03]	1.10
Conducive work environment (α = 0.62)	4.11 [3.97, 4.24]	0.07 [−0.02, 0.16]	−0.03 [−0.11, 0.06]	0.00 [−0.09, 0.08]	−0.36
I make sure there is sufficient light at my workstation.	5.40 [5.32, 5.48]	0.06 [−0.03, 0.15]	−0.07 [−0.15, 0.01]	−0.06 [−0.15, 0.03]	−2.02
I wear comfortable clothes.	5.37 [5.29, 5.45]	**0.14** [0.05, 0.22]	−0.06 [−0.15, 0.02]	**−0.10** [−0.19, −0.01]	−1.19
I regularly air the room.	4.98 [4.88, 5.09]	0.03 [−0.06, 0.11]	−0.05 [−0.13, 0.03]	0.00 [−0.09, 0.10]	−1.94
I use a setup that is technically close to the setup at my on-site workstation.	4.84 [4.72, 4.96]	**0.15** [0.06, 0.25]	−0.07 [−0.16, 0.02]	0.06 [−0.02, 0.14]	−0.36
I set up a conducive work environment.	4.33 [4.21, 4.45]	**0.16** [0.07, 0.25]	−0.07 [−0.15, 0.01]	0.06 [−0.02, 0.15]	0.30
*I listen to music that helps me concentrate.*	4.22 [4.06, 4.37]	0.05 [−0.04, 0.14]	**0.10** [0.01, 0.18]	−0.04 [−0.12, 0.05]	−0.74
I configure my workstation ergonomically.	4.08 [3.93, 4.23]	0.02 [−0.07, 0.12]	−0.04 [−0.12, 0.04]	−0.01 [−0.09, 0.08]	−0.89
I set up a pleasant room climate.	3.82 [3.68, 3.95]	0.06 [−0.03, 0.15]	0.03 [−0.05, 0.12]	−0.02 [−0.10, 0.07]	−0.18
I reduce potential sources of distraction by placing them out of reach of my workstation.	2.88 [2.75, 3.02]	0.06 [−0.03, 0.15]	−0.02 [−0.11, 0.06]	0.00 [−0.09, 0.08]	0.91
I personalize my workstation.	2.71 [2.56, 2.86]	−0.03 [−0.12, 0.05]	−0.02 [−0.11, 0.07]	0.07 [−0.02, 0.15]	0.11
I try to reduce distraction factors.	2.57 [2.45, 2.70]	**0.12** [0.03, 0.21]	−0.01 [−0.09, 0.08]	0.00 [−0.09, 0.08]	2.00
Temporal (α = 0.66)	3.61 [3.46, 3.76]	0.03 [−0.06, 0.12]	−0.01 [−0.09, 0.08]	−0.03 [−0.12, 0.06]	−0.23
Temporal structure (α = 0.80)	3.65 [3.50, 3.81]	0.05 [−0.04, 0.15]	0.01 [−0.07, 0.10]	−0.05 [−0.13, 0.04]	−0.03
I have a set time routine to start the workday in the morning.	4.80 [4.68, 4.92]	0.02 [−0.07, 0.10]	−0.05 [−0.13, 0.03]	**−0.09** [−0.17, 0.00]	−1.82
I structure my workday temporarily.	4.51 [4.39, 4.63]	**0.18** [0.08, 0.27]	−0.07 [−0.16, 0.01]	−0.07 [−0.16, 0.01]	0.26
I schedule in advance when I will work in my home office and when I will work on-site.	4.36 [4.22, 4.51]	0.09 [−0.01, 0.18]	0.00 [−0.09, 0.09]	0.02 [−0.06, 0.11]	−0.54
I log my working hours.	4.30 [4.13, 4.48]	**0.15** [0.06, 0.24]	0.08 [−0.01, 0.17]	−0.05 [−0.14, 0.04]	0.24
I strictly separate my work time from my leisure time.	4.07 [3.94, 4.20]	**0.22** [0.12, 0.31]	0.00 [−0.08, 0.09]	**−0.14** [−0.22, −0.05]	1.23
I have set days for working from home.	3.75 [3.58, 3.93]	0.02 [−0.07, 0.11]	−0.02 [−0.11, 0.06]	0.01 [−0.07, 0.10]	−0.52
I take a regular lunch break at set times.	3.70 [3.56, 3.85]	−0.03 [−0.12, 0.06]	−0.03 [−0.12, 0.05]	−0.03 [−0.11, 0.06]	−1.03
I have a set time routine for the transition from work to leisure.	3.38 [3.23, 3.53]	0.02 [−0.07, 0.11]	0.01 [−0.08, 0.09]	**−0.10** [−0.19, −0.02]	−0.13
I do not work beyond my working hours agreed with the employer.	3.15 [3.00, 3.29]	−0.02 [−0.11, 0.07]	**0.13** [0.05, 0.22]	−0.02 [−0.10, 0.07]	−0.20
I align my break schedule with the official break times of my organization.	2.82 [2.65, 2.99]	0.07 [−0.03, 0.16]	0.01 [−0.08, 0.10]	−0.04 [−0.13, 0.05]	1.07
I strictly adhere to set working hours.	2.64 [2.50, 2.78]	−0.01 [−0.10, 0.09]	0.05 [−0.04, 0.14]	−0.08 [−0.16, 0.01]	0.50
I schedule regular breaks.	2.29 [2.17, 2.41]	−0.04 [−0.13, 0.05]	0.03 [−0.06, 0.11]	0.02 [−0.07, 0.10]	0.56
Temporal flexibility (α = 0.56)	3.54 [3.40, 3.68]	−0.01 [−0.10, 0.08]	−0.03 [−0.12, 0.05]	0.00 [−0.08, 0.09]	−0.57
I am temporally flexible in handling urgent work requests.	4.79 [4.68, 4.90]	0.08 [−0.01, 0.18]	**−0.10** [−0.19, −0.01]	0.04 [−0.05, 0.12]	−1.10
I schedule my work time in order to get the most of my leisure time.	3.97 [3.85, 4.10]	−0.02 [−0.11, 0.06]	0.01 [−0.07, 0.10]	−0.01 [−0.10, 0.07]	−1.27
If my work is short on time, I’ll “save it up” to make up for it in the next days.	3.65 [3.50, 3.79]	−0.05 [−0.14, 0.04]	−0.04 [−0.12, 0.05]	**−0.10** [−0.18, −0.01]	−1.20
I schedule my leisure time in order to get the most of my work time.	3.49 [3.36, 3.62]	**0.16** [0.08, 0.25]	**−0.08** [−0.16, 0.00]	0.00 [−0.08, 0.08]	1.33
I flexibly transfer personal matters to times when I typically work.	3.17 [3.04, 3.30]	**−0.15** [−0.24, −0.06]	−0.01 [−0.10, 0.07]	0.01 [−0.07, 0.10]	−1.74
If my leisure is short on time, I’ll “save it up” to make up for it in the next days.	3.00 [2.87, 3.13]	0.00 [−0.08, 0.09]	0.02 [−0.06, 0.11]	−0.03 [−0.12, 0.06]	0.18
I flexibly transfer my work to times when I typically attend to personal matters.	2.68 [2.55, 2.81]	−0.07 [−0.17, 0.04]	−0.04 [−0.14, 0.05]	**0.09** [0.00, 0.18]	−0.19
Behavioral (α = 0.83)	3.87 [3.73, 4.01]	**0.10** [0.01, 0.19]	0.00 [−0.09, 0.08]	−0.03 [−0.11, 0.06]	0.19
Behavioral separation of work and leisure (α = 0.84)	3.69 [3.54, 3.85]	0.08 [−0.02, 0.17]	0.02 [−0.07, 0.10]	−0.06 [−0.14, 0.03]	0.15
I have a technological routine for the transition into work at the start of the work day.	5.11 [5.00, 5.22]	**0.15** [0.05, 0.24]	0.03 [−0.05, 0.12]	−0.08 [−0.16, 0.00]	−0.74
I have a set technological routine facilitating the transition from work to leisure.	4.89 [4.76, 5.02]	**0.14** [0.04, 0.25]	0.06 [−0.04, 0.15]	−0.03 [−0.12, 0.06]	−0.56
I maintain the same routines of my on-site work.	4.21 [4.09, 4.34]	**0.24** [0.15, 0.33]	0.02 [−0.06, 0.10]	**−0.22** [−0.30, −0.13]	1.32
I avoid reading non-work related materials at work.	3.88 [3.75, 4.01]	**0.15** [0.06, 0.25]	−0.01 [−0.09, 0.07]	−0.02 [−0.10, 0.06]	0.76
I attend to personal matters at work only when taking a break or during lunch hour.	3.82 [3.69, 3.96]	**0.17** [0.08, 0.26]	0.03 [−0.05, 0.12]	−0.06 [−0.15, 0.02]	1.00
I do not take work-related calls after hours.	3.73 [3.57, 3.89]	−0.01 [−0.10, 0.09]	0.03 [−0.06, 0.12]	−0.06 [−0.14, 0.03]	−0.80
I do not respond to work-related messages after hours.	3.62 [3.46, 3.78]	0.01 [−0.09, 0.11]	0.01 [−0.08, 0.10]	**−0.14** [−0.23, −0.05]	−0.48
I have a rule which leisure aspects are allowed to spill over into work and which not.	3.48 [3.33, 3.63]	**0.13** [0.04, 0.22]	0.02 [−0.06, 0.11]	0.09 [0.00, 0.17]	1.02
I have a rule which work aspects are allowed to spill over into leisure and which not.	3.47 [3.32, 3.62]	0.08 [−0.01, 0.17]	−0.02 [−0.11, 0.06]	−0.02 [−0.11, 0.07]	0.47
I do not go back to work after hours.	3.45 [3.31, 3.60]	0.03 [−0.06, 0.12]	0.04 [−0.05, 0.12]	−0.09 [−0.17, 0.00]	−0.07
I use breaks to strictly separate work time from leisure time.	3.32 [3.19, 3.46]	−0.03 [−0.12, 0.06]	−0.02 [−0.11, 0.06]	−0.02 [−0.11, 0.06]	−0.59
I do not read work-related messages after hours.	3.27 [3.11, 3.43]	0.03 [−0.07, 0.12]	0.00 [−0.09, 0.09]	**−0.14** [−0.23, −0.05]	0.10
I have set rituals facilitating the transition from work to leisure.	3.25 [3.10, 3.39]	0.04 [−0.05, 0.13]	0.03 [−0.05, 0.12]	−0.05 [−0.14, 0.03]	0.26
I avoid talking about work-related matters in leisure contexts.	2.98 [2.85, 3.10]	0.01 [−0.08, 0.11]	−0.02 [−0.11, 0.07]	−0.05 [−0.14, 0.04]	0.33
I avoid talking about personal matters in work contexts.	2.89 [2.77, 3.01]	0.00 [−0.09, 0.09]	0.02 [−0.06, 0.11]	0.03 [−0.05, 0.12]	0.29
Conducive work attitude (α = 0.65)	4.44 [4.32, 4,56]	**0.19** [0.10, 0.27]	−0.03 [−0.11, 0.05]	−0.02 [−0.10, 0.07]	0.46
I value the benefits of working from home.	5.28 [5.18, 5.38]	**0.13** [0.04, 0.22]	**−0.08** [−0.17, 0.00]	0.00 [−0.08, 0.08]	−1.15
I get organized at work.	4.98 [4.88, 5.09]	**0.15** [0.06, 0.24]	−0.04 [−0.12, 0.04]	0.02 [−0.06, 0.10]	−0.58
I show a particularly high level of dedication.	4.78 [4.69, 4.88]	**0.43** [0.35, 0.51]	−0.05 [−0.12, 0.03]	−0.01 [−0.09, 0.07]	2.69
I try to strengthen my supervisor’s confidence in the quality of my work.	4.78 [4.67, 4.88]	**0.26** [0.17, 0.35]	0.03 [−0.06, 0.11]	0.02 [−0.06, 0.11]	0.86
I adjust my attitude and behavior to optimally focus and concentrate at work.	4.70 [4.61, 4.80]	**0.31** [0.22, 0.39]	−0.05 [−0.12, 0.03]	−0.06 [−0.14, 0.02]	1.48
I schedule tasks that can be done particularly well at home.	4.61 [4.47, 4.74]	0.05 [−0.04, 0.14]	0.01 [−0.08, 0.10]	−0.06 [−0.15, 0.02]	−1.25
I set personal daily goals at work.	4.44 [4.32, 4.56]	**0.15** [0.06, 0.24]	−0.04 [−0.13, 0.04]	**−0.09** [−0.18, −0.01]	0.06
I take a short lunch break and quickly continue working to get done as much as possible.	3.31 [3.17, 3.44]	0.06 [−0.03, 0.14]	−0.02 [−0.10, 0.07]	−0.04 [−0.13, 0.04]	0.39
I practice self-praise.	3.06 [2.92, 3.20]	**0.14** [0.05, 0.23]	−0.03 [−0.11, 0.06]	0.07 [−0.02, 0.16]	1.63
Health-promoting measures (α = 0.61)	3.39 [3.25, 3.53]	0.01 [−0.08, 0.10]	−0.02 [−0.10, 0.06]	0.03 [−0.05, 0.12]	−0.22
I pay attention to healthy eating.	4.27 [4.15, 4.39]	**0.13** [0.04, 0.21]	0.00 [−0.08, 0.08]	0.00 [−0.08, 0.09]	0.03
I adapt my work day to my bio-rhythm.	3.74 [3.61, 3.87]	0.03 [−0.06, 0.12]	−0.01 [−0.09, 0.08]	0.03 [−0.05, 0.12]	−0.38
I integrate exercise into my work day.	3.51 [3.38, 3.64]	0.02 [−0.07, 0.11]	0.00 [−0.08, 0.08]	0.02 [−0.06, 0.11]	−0.24
I integrate outdoor activities into my work day.	3.44 [3.30, 3.58]	−0.01 [−0.10, 0.07]	−0.01 [−0.09, 0.07]	**0.09** [0.01, 0.18]	−0.51
I regularly take a “power nap”.	1.96 [1.83, 2.08]	**−0.13** [−0.21, −0.04]	−0.08 [−0.17, 0.00]	0.01 [−0.07, 0.10]	0.01
Communicative (α = 0.78)	4.03 [3.89, 4.17]	**0.12** [0.03, 0.21]	−0.03 [−0.12, 0.06]	−0.01 [−0.09, 0.08]	0.29
Make arrangements (α = 0.82)	3.54 [3.39, 3.70]	0.09 [0.00, 0.18]	−0.03 [−0.12, 0.06]	−0.01 [−0.10, 0.08]	0.52
I make arrangements with household members facilitating undisturbed work.	4.51 [4.38, 4.64]	**0.17** [0.08, 0.26]	0.04 [−0.05, 0.12]	0.01 [−0.08, 0.10]	0.22
I make arrangements with colleagues/supervisors/my employer about when I can(not) be reached regarding work.	4.18 [4.04, 4.33]	0.08 [−0.01, 0.17]	−0.07 [−0.16, 0.01]	**−0.10** [−0.18, −0.01]	−0.35
I’ll confront household members if agreements about work and leisure are violated.	3.79 [3.64, 3.95]	0.07 [−0.03, 0.16]	0.00 [−0.09, 0.09]	0.05 [−0.04, 0.14]	−0.09
I make arrangements with customers/clients about when I can(not) be reached regarding work.	3.66 [3.50, 3.82]	0.05 [−0.04, 0.14]	−0.04 [−0.13, 0.05]	−0.01 [−0.10, 0.07]	−0.07
I’ll confront colleagues/supervisors/my employer if agreements about work and leisure are violated.	3.04 [2.89, 3.19]	**0.15** [0.06, 0.24]	−0.03 [−0.12, 0.06]	0.00 [−0.10, 0.09]	1.75
Household members make arrangements with me in order to limit my workload.	2.79 [2.64, 2.93]	0.04 [−0.05, 0.13]	−0.04 [−0.13, 0.05]	0.00 [−0.09, 0.09]	0.85
I’ll confront clients/customers if agreements about work and leisure matters are violated.	2.61 [2.45, 2.77]	0.07 [−0.03, 0.17]	−0.03 [−0.13, 0.06]	0.03 [−0.06, 0.13]	1.35
Keep connection (α = 0.60)	4.46 [4.32, 4.59]	**0.15** [0.06, 0.24]	−0.03 [−0.12, 0.05]	0.00 [−0.09, 0.08]	0.05
I use various communication channels.	5.54 [5.47, 5.62]	**0.17** [0.08, 0.27]	0.03 [−0.06, 0.11]	−0.04 [−0.12, 0.04]	−0.97
I keep connected via technology to respond to colleagues/supervisors/my employer/customers/clients quickly.	5.42 [5.34, 5.50]	**0.20** [0.11, 0.29]	−0.01 [−0.09, 0.08]	0.04 [−0.05, 0.12]	−0.57
I make small talk with my colleagues/supervisors/my employer.	4.63 [4.52, 4.74]	**0.09** [0.00, 0.17]	−0.07 [−0.16, 0.02]	−0.03 [−0.11, 0.05]	−0.84
I communicate expectations and work progress with colleagues/supervisors/my employer.	4.20 [4.08, 4.33]	**0.21** [0.12, 0.29]	−0.05 [−0.14, 0.03]	0.03 [−0.05, 0.11]	0.96
I use modern communication technology with colleagues/supervisors/my employer such as instant messaging.	4.14 [3.97, 4.30]	**0.14** [0.05, 0.23]	**−0.10** [−0.19, −0.02]	0.03 [−0.05, 0.11]	0.32
I seek social interaction after work.	3.70 [3.58, 3.83]	**0.14** [0.05, 0.23]	0.05 [−0.04, 0.13]	−0.05 [−0.13, 0.03]	0.86
I use technology to stay in personal contact with colleagues.	3.54 [3.38, 3.69]	**0.10** [0.01, 0.19]	−0.05 [−0.14, 0.03]	−0.01 [−0.09, 0.08]	0.59

T, telework strategy; BMP, boundary management preferences; TE, telework experience. β_T_ represents the main effect of the telework strategy on job performance. β_T x BMP_ represents the interaction effect between the telework strategy and boundary management preferences on job performance. β_T x TE_ represents the interaction effect between the telework strategy and telework experience on job performance. Δ*M*, β_T_ is based on the difference between the *z*-scaled mean implementation (*M*) and the *z*-scaled beta-coefficient on job performance (β_T_) of the telework strategy. *M*, β_T_, β_T x BMP_, and β_T x TE_ are reported with 95%-confidence intervals. Telework strategies in italics were recoded. Multiple regression results were controlled for age, gender, living space, and living with children. Results in bold are significant at the *p* ≤ 0.05 level.

#### 2.2.2. Job performance

Participants were instructed that the assessment of job performance refers to their job performance when teleworking. Self-reported job performance (α = 0.70, *M* = 4.13, *SD* = 0.52) was assessed using three items translated into German (“How would you rate your job performance as an individual employee?”, “Think about your most recent assessment of your job performance or the most recent time you received feedback from your supervisor. How do you think your supervisor would rate your performance?”, “How would you rate your performance as a work team member?”; Bal and De Lange, 2015). Participants responded on a 5-point Likert scale ranging from 1 (*very poor*) to 5 (*excellent*). Participants could indicate “not applicable” on the item referring to their team performance. Nineteen participants aborted the survey before reporting their job performance leading to missing values. Even though self-reported job performance measures have limitations they substantially overlap with supervisor ratings (e.g., Heidemeier and Moser, 2009).

#### 2.2.3. Boundary management preferences

Inspired by Kossek et al. (2006), we presented participants the following prompt translated into German: “With the increasing demands of work and home, employees may work in different ways to handle these demands.” We then measured gradual interindividual differences in boundary management preferences with the item: “All in all, do you currently see yourself as someone who tries to keep work and personal roles separated most of the time or someone who tries to keep them integrated?” Participants responded on a 6-point Likert scale ranging from 1 (*I prefer to separate the roles*) to 6 (*I prefer to integrate the roles*). We recoded responses so that higher scores indicate preferences for separation (*M* = 3.76, *SD* = 1.67). Sixteen participants aborted the online survey before reporting their boundary management preferences leading to missing values. In the following, we use the terms *integrators*/*separators* to refer to individuals relatively lower/higher on the continuous dimension of boundary management preferences.

#### 2.2.4. Telework experience

Gradual interindividual differences in telework experience (*M* = 4.08, *SD* = 1.67) were assessed with, the item “How experienced are you with teleworking?” translated into German. Participants responded on a 6-point Likert scale ranging from 1 (*completely unexperienced*) to 6 (*completely experienced*). Twenty-four participants aborted the online survey before reporting their telework experience leading to missing values. In the following, we use the terms *unexperienced*/*experienced teleworkers* to refer to individuals relatively lower/higher on the continuous dimension of telework experience.

### 2.3. Analytical approach

We used the statistical program R (version 4.1.0; R Core Team, 2018) and the interface RStudio (version 1.2.5042; RStudio Team, 2016) for all analyses. The data and statistical code can be found in the Online Supplement at https://osf.io/gqpdf/. To identify broader patterns of results on a higher aggregated level, we summarized all appropriate results for super- and subordinate categories of telework strategies by computing (weighted) means.

To answer the question of how much telework strategies are implemented, we computed the means of the individual implementation of each of the 85 telework strategies. We then computed 85 multiple linear regressions of the individual implementation of each telework strategy on job performance. We included boundary management preferences and telework experience as additional predictors in each multiple linear regression to examine their interaction effects with telework strategies on job performance. We also included control variables (e.g., Binnewies et al., 2020; Troll et al., 2022), that is, basic demographic (age, gender) and situational aspects (living space in m^2^, *M* = 109.87, *SD* = 50.16; living with children, *M* = 0.25, *SD* = 0.43). Job performance and all predictor variables were *z*-scaled, except for the dummy-coded variables gender (0/1 = female/male) and living with children (0/1 = no/yes). We answer the question of how the individual implementation of telework strategies is associated with job performance based on the β-coefficients of the telework strategies on job performance. We answer the questions of the moderation effects of boundary management preferences and telework experience, respectively, on the association between telework strategies and job performance based on the β-coefficients corresponding to these interaction effects in the multiple linear regressions.

To examine how the telework strategies’ average implementation is associated with the telework strategies’ association with job performance, we correlated the 85 means of the implementation of the telework strategies with the 85 β-coefficients of the telework strategies on job performance in the outlined multiple linear regressions. To answer the question of how the telework strategies’ relative implementation diverges from their relative association with job performance, we computed differences between a telework strategy’s *z*-scaled implementation and a telework strategy’s *z*-scaled β-coefficient on job performance in the outlined multiple linear regressions.

## 3. Results

The results of all research questions except RQ 3.1 are summarized in Table 1 (see Supplementary Material Section A for a corresponding table including information on standard deviations of the implementation of telework strategies, bivariate correlations between telework strategies and job performance, further regression coefficients (intercept, boundary management preferences, telework experience, and control variables), *R*^2^, *R*^2^_Adjusted_, and results of the *F*-test; https://osf.io/gqpdf/).

Concerning the implementation of telework strategies (RQ 1), we found communicative telework strategies on average to be the most implemented ( $\bar{M}$ = 4.03 [3.89, 4.17]), followed by physical ( $\bar{M}$ = 3.95 [3.80, 4.10]), behavioral ( $\bar{M}$ = 3.87 [3.73, 4.01]), and temporal ( $\bar{M}$ = 3.61 [3.46, 3.76]) telework strategies. Zooming-in on the level of subcategories, telework strategies related to keep connection ( $\bar{M}$ = 4.46 [4.32, 4.59]) and to conducive work attitude ( $\bar{M}$ = 4.44, [4.32, 4.56]) were on average most implemented and more implemented than the telework strategies related to all remaining subcategories. To facilitate quickly grasping which specific telework strategies drive these effects, we ordered the telework strategies in Table 1 by the mean of implementation (from high to low) in their respective subcategory.

Concerning associations between the individual implementation of telework strategies and job performance (RQ 2), we found communicative ( $\bar{\beta }$ = 0.12 [0.03, 0.21]) and behavioral ( $\bar{\beta }$ = 0.10 [0.01, 0.19]) telework strategies on average to be positively associated. The average performance associations of telework strategies of the respective subcategories allow us to paint a more differentiated picture: Telework strategies related to keep connection ( $\bar{\beta }$ = 0.15 [0.06, 0.24]; 7 of 7 composing telework strategies had significant βs) and to conducive work attitude ( $\bar{\beta }$ = 0.19 [0.10, 0.27]; 7 of 9 composing telework strategies had significant βs) were on average positively associated with job performance driving the positive performance association of communicative and behavioral telework strategies, respectively. This pattern of results remained robust when applying alternative analytical approaches, that is, computing (factor analytically identified) scales for telework strategy subcategories averaging the implementation of the respective composing telework strategies per participant and simultaneously entering these scales into multiple linear regressions predicting job performance (see Supplementary Material Section B and Section C at https://osf.io/gqpdf/).

Concerning the association between the telework strategies’ average implementation and association with job performance (RQ 3.1), we found a positive correlation between the 85 means of the implementation of the telework strategies and the 85 β-coefficients of the telework strategies on job performance (*r* = 0.55 [0.39, 0.69], *t*(83) = 6.06, *p* < 0.001). Concerning the divergence between the telework strategies’ relative implementation from their relative association with job performance (RQ 3.2), we found positive averaged differences between a telework strategy’s *z*-scaled implementation and a telework strategy’s *z*-scaled β-coefficient on job performance for communicative ( $\bar{\Delta }$ = 0.29) and behavioral ( $\bar{\Delta }$ = 0.19) telework strategies, indicating that these categories’ telework strategies in average had relative associations with job performance exceeding their relative implementations. As communicative and behavioral telework strategies were on average positively associated with job performance, the related telework strategies might be on average considered under implemented. The higher resolved level of subcategories allows us to draw more nuanced inferences: Telework strategies related to keep connection and to conducive work attitude were the only subcategories on average positively associated with job performance, and thus, of most interest when identifying under implemented telework strategies. Here, we found telework strategies related to conducive work attitude showing on average large positive differences ( $\bar{\Delta }$ = 0.46), indicating that particularly these telework strategies might be seen as under implemented, whereas telework strategies related to keep connection had on average substantially less positive differences ( $\bar{\Delta }$ = 0.05). In comparison, we found the most negative averaged differences for temporal telework strategies ( $\bar{\Delta }$ = −0.23), driven by telework strategies related to temporal flexibility ( $\bar{\Delta }$ = −0.57), indicating that the related telework strategies on average had relative associations with job performance subceeding their relative implementations and might thus be considered over implemented.

Concerning the moderation effects of boundary management preferences (RQ 4.1) on the association between telework strategies and job performance, we found a positive interaction effect for a telework strategy related to temporal structure (“I do not work beyond my working hours agreed with the employer.”, β = 0.13 [0.05, 0.22], *p* = 0.002), indicating that this telework strategy might be more suitable for separators. We found negative interaction effects for specific telework strategies related to temporal flexibility (“I am temporarily flexible in handling urgent work requests.”, β = −0.10 [−0.19, −0.01], *p* = 0.025; “I schedule my leisure time in order to get the most of my work time.”, β = −0.08 [−0.16, 0.00], *p* = 0.050), keep connection (“I use modern communication technology with colleagues/supervisors/my employer such as instant messaging.”, β = −0.10 [−0.19, −0.02], *p* = 0.014), conducive work environment (“I listen to music that helps me concentrate.”, β = 0.10 [0.01, 0.18], *p* = 0.028, this telework strategy was recoded so that the interaction effect needs to be reversed), and conducive work attitude (“I value the benefits of working from home.”, β = −0.08 [−0.17, 0.00], *p* = 0.050), indicating that these telework strategies might be more suitable for integrators.

Concerning the moderation effects of telework experience (RQ 4.2) on the association between telework strategies and job performance, we found positive interaction effects for specific telework strategies related to physical separation of work and leisure (“I regularly work outside my home.”, β = −0.12 [−0.22, −0.02], *p* = 0.015, this telework strategy was recoded so that the interaction effect needs to be reversed), health-promoting measures (“I integrate outdoor activities into my work day.”, β = 0.09 [0.01, 0.18], *p* = 0.031), and temporal flexibility (“I flexibly transfer my work to times when I typically attend to personal matters.”, β = 0.09 [0.00, 0.18], *p* = 0.044), indicating that these telework strategies might be more suitable for experienced teleworkers. We found negative interaction effects for specific telework strategies related to behavioral separation of work and leisure (“I maintain the same routines of my on-site work.”, β = −0.22 [−0.30, −0.13], *p* < 0.001; “I do not read work-related messages after hours.”, β = −0.14 [−0.23, −0.05], *p* = 0.002; “I do not respond to work-related messages after hours.,” β = −0.14 [−0.23, −0.05], *p* = 0.002), temporal structure (“I strictly separate my work time from my leisure time.”, β = −0.14 [−0.22, −0.05], *p* = 0.001; “I have a set time routine for the transition from work to leisure.”, β = −0.10 [−0.19, −0.02], *p* = 0.016; “I have a set time routine to start the workday in the morning.”, β = −0.09 [−0.17, 0.00], *p* = 0.043), conducive work environment (“I wear comfortable clothes.”, β = −0.10 [−0.19, −0.01], *p* = 0.031), make arrangements (“I make arrangements with colleagues/supervisors/my employer about when I can (not) be reached regarding work.”, β = −0.10 [−0.18, −0.01], *p* = 0.028), temporal flexibility (“If my work is short on time, I’ll ‘save it up’ to make up for it in the next days.”, β = −0.10 [−0.18, −0.01], *p* = 0.035), conducive work attitude (“I set personal daily goals at work.”, β = −0.09 [−0.18, −0.01], *p* = 0.027), and physical separation of work and leisure (“I arrange my workstation visually like a typical office.”, β = −0.09 [−0.18, 0.00], *p* = 0.041), indicating that these telework strategies might be more suitable for unexperienced teleworkers.

Following up on this, we explored whether the telework strategies with significant interaction effects on job performance were implemented more by the group of teleworkers the interaction effect was in favor of (see Table 2). We therefore divided the sample into separators versus integrators and experienced versus unexperienced teleworkers, respectively, and computed two-sample *t*-tests concerning the implementation of the telework strategies with significant interaction effects. We divided the sample using the respective scale centers (3.50) as cut-off values. This led to a group of separators (*n* = 288, 54%) ranking themselves closer to the scale anchor indicating a preference for separation (> 3.50) versus a group of integrators (*n* = 244, 46%) ranking themselves closer to the scale anchor indicating a preference for integration (< 3.50). Likewise, we divided the sample into a group of experienced teleworkers (*n* = 348, 66%) ranking themselves closer to the scale anchor indicating high telework experience (> 3.50) versus a group of unexperienced teleworkers (*n* = 176, 34%) ranking themselves closer to the scale anchor indicating low telework experience (< 3.50). Of the 20 significant interaction effects, we found higher implementations by the group of teleworkers the respective interaction effect was in favor of for six interaction effects, whereas we found significant lower implementations by the group of teleworkers the respective interaction effect was in favor of for two interaction effects.

**Table 2:** Mean value differences of the implementation of telework strategies with significant interaction effects on job performance for boundary management preferences and telework experience.

Telework strategies for boundary management preferences	Separators	Integrators		
	*M*	*SD*	*M*	*SD*	*𝞓M*	*t*	*df*	*p*
I do not work beyond my working hours agreed with the employer.	3.47	1.69	2.76	1.65	**0.70**	**4.80**	521	<0.001
I value the benefits of working from home.	5.34	1.17	5.22	1.21	0.12	1.16	525	0.25
I use modern communication technology with colleagues/supervisors/my employer such as instant messaging.	4.17	1.93	4.08	1.93	0.10	0.56	512	0.58
*I listen to music that helps me concentrate.*	4.19	1.87	4.23	1.86	−0.04	−0.25	525	0.80
I schedule my leisure time in order to get the most of my work time.	3.36	1.56	3.66	1.43	**−0.30**	**−2.29**	521	0.022
I am temporally flexible in handling urgent work requests.	4.60	1.34	5.06	1.06	**−0.46**	**−4.37**	523	<0.001
Telework strategies for telework experience	Experienced	Unexperienced		
	*M*	*SD*	*M*	*SD*	*ΔM*	*t*	*df*	*p*
I arrange my workstation visually like a typical office.	3.91	1.82	3.24	1.81	**0.67**	**3.95**	521	<0.001
I flexibly transfer my work to times when I typically attend to personal matters.	2.82	1.55	2.38	1.40	**0.44**	**3.16**	517	0.002
I set personal daily goals at work.	4.54	1.35	4.27	1.51	**0.27**	**2.10**	516	0.037
I integrate outdoor activities into my work day.	3.52	1.66	3.28	1.69	0.23	1.51	522	0.13
If my work is short on time, I’ll “save it up” to make up for it in the next days.	3.70	1.63	3.55	1.76	0.15	0.92	471	0.36
I make arrangements with colleagues/supervisors/my employer about when I can and cannot be reached regarding work matters.	4.22	1.69	4.13	1.71	0.09	0.58	511	0.56
I wear comfortable clothes.	5.37	0.92	5.35	1.00	0.02	0.23	515	0.82
I maintain the same routines of my on-site work.	4.24	1.50	4.23	1.45	0.01	0.09	509	0.93
I have a set time routine to start the workday in the morning.	4.78	1.46	4.84	1.39	−0.05	−0.40	522	0.69
I strictly separate my work time from my leisure time.	4.04	1.53	4.19	1.56	−0.15	−1.08	522	0.28
I have a set time routine for the transition from work to leisure.	3.33	1.77	3.47	1.78	−0.15	−0.90	519	0.37
*I regularly work outside my home.*	5.32	1.17	5.58	0.96	**−0.26**	**−2.72**	415	0.007
I do not read work-related messages after hours.	3.17	1.90	3.49	1.96	−0.32	−1.80	521	0.073
I do not respond to work-related messages after hours.	3.46	1.91	3.94	1.84	**−0.48**	**−2.76**	521	0.006

Δ*M*, mean value difference of the implementation of the telework strategy between separators and integrators/experienced and unexperienced teleworkers. The sample was divided into separators (*n* = 288, 54%) versus integrators (*n* = 244, 46%) using the scale center (3.50) of the variable boundary management preferences as cut-off value. The sample was divided into experienced (*n* = 348, 66%) versus unexperienced (*n* = 176, 34%) teleworkers using the scale center (3.50) of the variable telework experience as cut-off value. Telework strategies in italics were recoded. Telework strategies sorted by Δ*M* in descending order. Δ*M* and *t*-values in bold are significant at the *p* ≤ 0.05 level.

## 4. Discussion

Drawing from previous research and the popular media, we examined a comprehensive set of 85 highly resolved telework strategies in a sample of 548 teleworkers. We found that the most implemented telework strategies tend to be the ones most positively associated with job performance (RQ 3.1). These serve less the purpose of drawing boundaries between work- and private-life (e.g., Fonner and Stache, 2012; Basile and Beauregard, 2016) but rather purposes of working task-oriented and productively (e.g., Greer and Payne, 2014) by adopting a conducive work attitude and of keeping social contact (e.g., Kowalski and Swanson, 2005; Turetken et al., 2011) by using modern communication technology (RQ 1, 2). Taking the level of implementation into account, teleworkers might be particularly advised to implement telework strategies related to conducive work attitude (RQ 3.2). In alignment with P-E fit (Kristof, 1996; Edwards, 2008) and boundary congruence/fit approaches (Kreiner, 2006; Ammons, 2013), we found that separators tend to benefit from telework strategies establishing boundaries between work- and private-life, whereas integrators tend to benefit from telework strategies dismantling boundaries between work- and private-life (RQ 4.1). Likewise, experienced teleworkers tend to benefit from telework strategies providing flexibility, whereas unexperienced teleworkers tend to benefit from telework strategies providing structure (RQ 4.2).

### 4.1. Implementation of telework strategies

We found telework strategies related to keep connection and to conducive work attitude to be most implemented. Aligning this result with previous research, Greer and Payne (2014) and Troll et al. (2022) observed similar patterns. Greer and Payne (2014) found telework strategies related to “be accessible” and to “communicate with coworkers/supervisor” to be among high performing teleworkers’ most frequently mentioned telework strategies, matching our finding of telework strategies related to keep connection. They also found telework strategies related to “adopt a work-oriented mindset”, “be extra productive”, “plan tasks”, and “set goals and prioritize” to be commonly mentioned, matching our finding of telework strategies related to conducive work attitude. Troll et al. (2022) found telework strategies related to modifying social conditions to be frequently implemented, but they laid a specific focus on getting motivated by friends/colleagues to work productively not matching the core of our telework strategies related to keep connection. However, Troll et al. (2022) also found telework strategies related to autonomous motivation (motivating oneself to start and endure work tasks) being frequently implemented that overlap with telework strategies related to conducive work attitude (e.g., practicing self-praise, showing dedication, reducing breaks to make progress).

It catches the eye that we found boundary related telework strategies to be less implemented than telework strategies related to keep connection and to conducive work attitude. This is remarkable because the largest proportion of research on telework strategies stems from boundary theory (Nippert-Eng, 1996; Ashforth et al., 2000) transferring boundary management strategies to the telework context (Tietze, 2002; Tietze and Musson, 2003; Myrie and Daly, 2009; Mustafa, 2010; Nansen et al., 2010; Fonner and Stache, 2012; Mustafa and Gold, 2013; Basile and Beauregard, 2016; Kossek, 2016; Allen et al., 2021; Haun et al., 2022). Also in the popular media (e.g., Cobler, n.d.; Stross, n.d.; Westdeutsche Zeitung, 2020) boundary related telework strategies receive much attention. Due to boundary related telework strategies being in the spotlight, one might be tempted to conclude that these are the most implemented. In contrast, the present study suggests that it is valuable to complement telework strategies serving other goals such as keeping social contact (Mann et al., 2000; Ilozor et al., 2001; Kowalski and Swanson, 2005; Golden and Raghuram, 2010; Turetken et al., 2011), and working task-oriented and productively (Greer and Payne, 2014; Troll et al., 2022) to paint a comprehensive picture of telework strategies’ implementation.

### 4.2. Associations between telework strategies and job performance

Responding to multiple calls for research on the effectiveness of boundary management strategies (Binnewies et al., 2020) and telework strategies (Allen et al., 2021; Rudolph et al., 2021), we found telework strategies related to conducive work attitude (driven by showing dedication, adjusting behavior and attitude to focus, strengthening the supervisor’s confidence in the own work quality, getting organized, setting goals, practicing self-praise, and valuing telework benefits) and to keep connection (driven by communicating expectations and work progress, keeping connected via technology, using various communication channels, seeking social interaction after work, using modern communication technology, and using technology to stay in personal contact with colleagues) being positively associated with job performance. These results fit with high performers’ implemented telework strategies: Greer and Payne (2014) found “adopt a work-oriented mindset”, “be extra productive”, “plan tasks”, and “set goals and prioritize” as well as “be accessible” and “communicate with coworkers/supervisor” to be commonly mentioned.^5^ Troll et al. (2022) found telework strategies related to autonomous motivation to predict job performance, matching our finding of a positive association between telework strategies related to conducive work attitude and job performance.^6^^,^^7^ Finally, our finding of telework strategies related to keep connection being positively associated with job performance might be aligned with MRT (Daft and Lengel, 1986) and goes well with Turetken et al.’s (2011) finding of communication media richness predicting teleworkers’ job performance.

We found telework strategies related to conducive work attitude and to keep connection to be positively associated with job performance, whereas boundary related telework strategies were less associated with job performance. On the one hand, this is in line with Kossek et al. (2006), who found the global implementation of boundary related telework strategies not being associated with job performance. On the other hand, this is striking because boundary related telework strategies are regularly referred to as “best-practice” (Golden, 2021) and proposed to foster productive teleworking in the popular media (e.g., Prophet, 2017; Schulz, 2020). The young literature on telework strategies might profit from complementing telework strategies serving goals of working task-oriented and productively, and keeping social contact when examining telework strategies’ impacts on work outcomes and deriving practical recommendations.

### 4.3. Divergences between telework strategies’ implementation and association with job performance

The present study is the first to quantitatively examine a large number of telework strategies on a highly resolved level, which enabled us to suggest that telework strategies more positively associated with job performance tend to be implemented more often. Thus, it seems that teleworkers have an intuitive understanding of the telework strategies important to job performance and tend to implement them accordingly. However, there were also telework strategies with substantial divergences in terms of their relative association with job performance and their relative implementation enabling us to derive initial fine-grained practical recommendations. We identified telework strategies related to conducive work attitude (driven by showing dedication, practicing self-praise, and adjusting attitude and behavior to focus) to be under implemented. In comparison, telework strategies related to keep connection were more implemented and less associated with job performance, leading to a smaller divergence. Thus, taking the current level of implementation into account, teleworkers might be advised to pay particular attention to implementing telework strategies related to conducive work attitude. Concerning telework strategies for which their relative association with job performance subceeded their relative implementation, we particularly found telework strategies related to temporal flexibility being over implemented (driven by transferring personal matters to work times, scheduling work time to get the most of leisure time, banking work times), so that teleworkers might be advised to reduce their implementation.

### 4.4. Moderating influences of boundary management preferences and telework experience

Following P-E fit approaches (preferences/needs-supply fit; Kristof, 1996; Edwards, 2008) and specific approaches related to boundary theory (Nippert-Eng, 1996; Ashforth et al., 2000), that is, the boundary congruence approach (Kreiner, 2006) and the boundary fit approach (Ammons, 2013), telework strategies congruent with individual boundary management preferences might be particularly beneficial to job performance as a fit between individual preferences and needs (boundary management preferences) and environmental boundary influences/boundary enactments (telework strategies) is achieved. Indeed, we found a telework strategy aligned to separating work- and private-life (i.e., not working beyond agreed hours) being more beneficial to job performance for separators. In comparison, we found telework strategies aligned to integrating work- and private-life (i.e., flexibly handling urgent work requests and scheduling leisure time to get the most of the work time) being more beneficial to job performance for integrators. We also identified three further telework strategies particularly beneficial to integrators that might align with a preference for integrating work- and private-life. First, using modern communication technology such as instant messaging might blur the borders between work- and private-life due to being continuously accessible for work matters. Second, listening to music that helps to concentrate might be seen as mingling a typical leisure activity with work. Third, valuing the benefits of telework might be particularly beneficial to integrators as many benefits of teleworking are aligned to a better integration of work- and private-life due to enhanced flexibility.

Concerning moderating influences of telework experience, we found two telework strategies related to spatial flexibility (i.e., working outside from home, for instance, in a café, and integrating outdoor activities into the workday) and one telework strategy related to temporal flexibility (i.e., flexibly transferring work to times when typically attending personal matters) being more beneficial to job performance for experienced teleworkers. In comparison, we found mainly telework strategies related to establishing routines (i.e., maintaining the routines of the on-site work, establishing routines to start and to end the work day, setting daily goals) and to adhering to work/non-work rules (i.e., strictly separating work and leisure time, not reading/responding to work messages after hours, making arrangements about when (not) to be reached regarding work) being more beneficial to job performance for unexperienced teleworkers. Thus, following the P-E fit approach (Kristof, 1996; Edwards, 2008), it seems that telework strategies related to flexibility might rather meet the preferences and needs of experienced teleworkers that might desire and be able to handle alternation resulting in a more positive association with job performance. In comparison, telework strategies related to establishing routines and work/non-work rules might rather meet the preferences and needs of unexperienced teleworkers that might desire and need structure resulting in a more positive association with job performance.

Overall, in the present study, we identified the effects of P-E fit on job performance, whereas previous research in the context of boundary congruence/fit (Kreiner, 2006; Ammons, 2013) rather focused on outcomes such as work-family-conflict, job satisfaction, commitment, and recovery (see Rothbard et al., 2005; Kreiner, 2006; Chen et al., 2009; Haun et al., 2022). The pattern of results underlined the utility of transferring P-E fit, in particular, boundary congruence/fit, to telework contexts to paint a differentiated picture of telework strategies’ effectiveness depending on teleworkers’ individual preferences and needs.

### 4.5. Theoretical implications

The present study sheds light on the puzzling impacts of individual telework strategies, an under-explored field of research that is not yet well-anchored in the scientific literature. Whereas most previous studies have focused on telework strategies aligned to establishing/dismantling boundaries between work- and private-life in the tradition of boundary theory (Nippert-Eng, 1996; Ashforth et al., 2000), the present results suggest that the young field of research on telework strategies might profit from expanding this narrow focus. In particular, we demonstrate that teleworkers rather tend to implement telework strategies serving goals such as working task-oriented and productively (e.g., Greer & Payne) as well as keeping social contact (e.g., Kowalski and Swanson, 2005). Even more so, these telework strategies were most decisive for job performance. Thus, future research on telework strategies could progress by adopting a broader focus on telework strategies serving divergent goals to understand more comprehensively telework strategies’ enigmatic impacts on various (tele-) work outcomes. The present study also contributes to the literature by demonstrating that applying the P-E fit framework (Kristof, 1996; Edwards, 2008) to the telework context helps to unravel the differential impacts of telework strategies when considering teleworkers’ individual preferences and needs. We did not find a one-fits-all solution to effective telework strategies uniformly applying to all teleworkers. The present findings rather suggest that the effectiveness of many telework strategies depends on teleworkers’ individual boundary management preferences and experience with working from home. Thus, marrying the literature streams of P-E fit, in particular boundary congruence/fit (Kreiner, 2006; Ammons, 2013), and telework strategies seems to be another promising avenue for future research to advance progress in this nascent research field.

### 4.6. Organizational implications

Whereas there are plenty of recommendations for implementing individual telework strategies spread throughout the popular media, the scientific literature still lags behind in providing empirical evidence on telework strategies’ effectiveness (Allen et al., 2021; Rudolph et al., 2021; see also Binnewies et al., 2020). The present study aims to fill this gap and delivers reassuring results on the implementation of commonly circulating telework strategies: In general, teleworkers seem to have an intuitive understanding of which telework strategies are effective. That is, teleworkers tend to implement telework strategies more often that are more positively associated with job performance. However, we still found telework strategies related to adopting a conducive work attitude (e.g., practicing self-praise) to be less implemented than they probably should be according to their strong association with job performance. If verified in future confirmatory research, organizations might pick up on these findings by taking measures to educate teleworkers about effective telework strategies, especially those that are yet poorly implemented. On the other side, we also identified telework strategies for which organizations might be advised to take measures to educate their teleworkers to implement them less. In particular, we found telework strategies related to temporal flexibility (e.g., transferring personal matters to work times) to be implemented more often than they probably should be based on their low or even negative association with job performance. However, organizations need to consider that the implementation of telework strategies may not always be a matter of choice. Real-life circumstances can occasionally hinder teleworkers from implementing effective strategies and from avoiding ineffective strategies. For instance, during the COVID-19 pandemic, many teleworkers have been affected by sudden school and daycare closures due to lockdown measures to limit the spread of the pandemic. Thus, many teleworkers might have had to switch flexibly between work requests and demands spilling over from their private-life (e.g., taking care of the children). Organizations might therefore also try to anticipate potential reasons (e.g., lack of childcare) for implementing less conducive telework strategies (e.g., transferring personal matters to work times) and to take action to mitigate these reasons (e.g., organizations might offer (virtual) childcare programs). Finally, organizations may adopt measures to identify groups of employees sharing common characteristics critical to the effectiveness of telework strategies and tailor advice (e.g., via organizational e-mail newsletters) or trainings educating about effective telework strategies to employees’ individual preferences and needs. This is particularly intriguing in situations such as the COVID-19 pandemic in which employees may be urged to telework considering themselves not prepared to do so (e.g., separators and unexperienced teleworkers).

### 4.7. Limitations and directions for future research

First, due to the cross-sectional design, we cannot draw causal inferences, that is, we can only demonstrate which telework strategies are associated with job performance, but this does not imply that the telework strategies cause between person differences in job performance. We thus suggest future research to apply longitudinal research designs (e.g., experience sampling/daily diary methods, Larson and Csikszentmihalyi, 2014; structural equation modeling approaches to cross-lagged panel models, Hamaker et al., 2015) to examine the directional impacts telework strategies and job performance have on one another over time. We also encourage future research to adopt (quasi-) experimental designs, for instance, to examine a training intervention in a pre-post control group design monitoring the implementation of telework strategies and job performance after the training (see also Rexroth et al., 2016; Binnewies et al., 2020). This could also demonstrate telework strategies’ trainability with practical implications for teleworkers that might be able to learn to telework productively by applying effective telework strategies. Against the background of ongoing change processes of work in the digital age, such as technological advances and increasing flexibility of working time and space, particularly online training interventions might be a promising starting point to do so (see Rexroth et al., 2017).

Second, our data is based on self-reports assessed at one measurement time point, which may have introduced common-method bias (CMB; Podsakoff et al., 2012). However, CMB does not always compromise results. For instance, in the present study, despite the large sample size, the majority of telework strategies did not show significant associations with job performance, which should have been the case, if CMB was a severe problem (Spector, 2006). Also CMB is less of a problem in regression models with multiple predictors and when testing interaction effects (Siemsen et al., 2010). Nevertheless, we suggest future research to assess telework outcomes such as job performance with multiple independent, objective indicators (e.g., supervisor/coworker ratings, customer satisfaction, objective records such as the number of claims processed). Also, telework strategies might be assessed through acquaintance reports (e.g., household members, coworkers). Measuring the implementation of telework strategies and telework outcomes at different time points would be a further approach to mitigate CMB.

Third, the directionality of boundary related telework strategies (see Hecht and Allen, 2009; Allen et al., 2014; Wepfer et al., 2018) might be considered, that is, telework strategies can either be geared toward keeping private matters out of work (versus integrating) or toward keeping work out of private matters (versus integrating). In our study, we summarized findings of telework strategies related to both boundary management directions as both indicate a separation/integration of work- and private-life. However, telework strategies with a different directionality might differentially impact work outcomes. Such a differentiated pattern might be masked because divergent effects might cancel each other out. Indeed, for some telework strategies, we observed a pattern that might provide initial support for this notion (however, this pattern does not apply uniformly to all concerned telework strategies requiring to draw conclusions with caution): We found rather positive performance associations for telework strategies geared to keeping private matters out of work (e.g., avoiding to read non-work related material at work, attending to personal matters only during breaks), whereas we found rather zero performance associations for telework strategies geared to keeping work out of private matters (e.g., not going back to work after hours, not reading/responding to work-related messages/calls after hours). Future research should consider telework strategies’ boundary management directionality to examine potential divergent effects on telework outcomes.

Fourth, the lack of associations of boundary related telework strategies with job performance does not imply that they do not have other positive effects. Quite the opposite, these telework strategies are likely to have various positive effects, particularly when it comes to outcomes such as reduced stress, well-being, and satisfaction (Binnewies et al., 2020; Haun et al., 2022). It should also be considered that telework strategies that have been found to be positively associated with job performance in the present study (e.g., scheduling leisure time to get the most out of work time) could have detrimental effects on other outcomes such as stress and well-being. Future research will profit from examining the effects of telework strategies on a broad set of telework outcomes to draw a differentiated picture of telework strategies’ various impacts.

Fifth, in the present study, we focused on boundary management preferences and telework experience as two important individual characteristics of teleworkers that had a moderating influence on the relationship between telework strategies and job performance. Future research could examine other individual characteristics that might affect the effectiveness of telework strategies. This would contribute to a more nuanced understanding of the puzzling effects of telework strategies on (tele-) work outcomes. For instance, whereas teleworkers’ personality traits such as extraversion and conscientiousness have already been shown to directly affect telework outcomes (O’Neill et al., 2009), little is known about their moderating effects. For example, it may be that extraverted teleworkers suffer particularly from social isolation (especially during times of pandemic), so they could benefit from implementing communicative telework strategies related to keep connection, such as engaging in virtual small talk with colleagues. Similarly, teleworkers with low conscientiousness might particularly benefit from telework strategies that help to maintain a clear daily structure, such as adhering to fixed work/non-work hours. However, these moderating factors are not limited to the individual characteristics of the teleworker but may also represent broader situational factors. For example, whereas we found that most boundary related telework strategies do not positively impact job performance, this could change when children are at home. In light of the lockdown measures during the COVID-19 pandemic, many teleworkers may have faced this situation. In these cases, for example, physical telework strategies related to physical separation of work and leisure, such as working in a separate room, could be beneficial because they may facilitate undisturbed work. A better understanding of the moderating factors that influence the relationship between telework strategies and (tele-) work outcomes could have immediate practical implications for organizations to tailor advice on telework strategies to employees’ individual characteristics and situational circumstances.

Sixth, in the present study, we applied a P-E fit approach (Kristof, 1996; Edwards, 2008) to a teleworker sample, which stands out due to teleworkers having great latitude to self-adjust various work environmental aspects (in contrast to stationary on-site workers). It might be an exciting future research topic to examine whether performance differences between teleworker and non-teleworker samples (Gajendran and Harrison, 2007) might (to some extent) be driven by enhanced P-E fit in telework contexts, in which teleworkers are empowered to self-adjust their working environment in a way that corresponds to their individual preferences and needs. Indeed, we found initial indications that teleworkers to some extent successfully customize their telework environment. For example, whereas we found separators to profit more from not working beyond their agreed working hours, we also found separators to implement this telework strategy more. Likewise, whereas we found integrators to profit more from being temporally flexible in handling urgent work requests and from scheduling their leisure time to get the most out of their work time, we also found integrators to implement these telework strategies more. Future confirmatory research could build on these exploratory findings.

Seventh, future research would profit from establishing a mutually accepted taxonomy of telework strategies by deriving theoretically sound dimensions of telework strategies and testing these with factor analytical or structural equation modeling procedures. A self-report questionnaire might be developed to measure the implementation of telework strategies meeting psychometric test properties. In particular, future research in this vein should make efforts to demonstrate that such a test actually measures the dimensions of telework strategies that it is claimed to measure, that is demonstrating construct validity (Badenes-Ribera et al., 2020). This would ensure that researchers use terms consistently and increase the comparability of research findings streamlining progress in this nascent research field.

Finally, we would like to emphasize the Northern European cultural context of our study, which is likely to have affected the individual ways in which participants organized their work processes from home. The cultural context has a strong influence on work-related values and norms (e.g., Hofstede, 1984; Schwartz, 1999), which also affect telework constellations (e.g., Peters and den Dulk, 2003; Adamovic, 2022). In addition, there have been cross-cultural differences in pandemic related measures to limit the spreading of the COVID-19 virus (e.g., imposing lockdowns; Bajaj et al., 2021) and in individuals’ psychological responses to the COVID-19 pandemic (Yap et al., 2021), which may have caused further cross-cultural differences in the adoption of telework strategies. We therefore suggest future research to address cross-cultural similarities and differences in the way teleworkers organize their work processes.

## 5. Conclusion

This study contributes to the young literature on telework strategies by demonstrating that extending a narrow focus on telework strategies stemming from boundary theory seems to be a fruitful avenue for research illuminating the puzzling impacts of the individual ways in which teleworkers organize their work processes. In particular, future research would profit from complementing telework strategies aligned to working task-oriented and productively by adopting a conducive work attitude and to keeping social contact by using modern communication technology. Also, taking a P-E fit perspective appears to be a promising approach to paint a more fine-grained picture of telework strategies’ differential impacts on work outcomes by taking teleworkers’ individual preferences and needs into account.

## Data availability statement

The data, codebook, R‑script, and supplementary results for this study are provided on the Open Science Framework and can be retrieved from the following link: https://osf.io/gqpdf/.

## Ethics statement

Ethical review and approval was not required for the study on human participants in accordance with the local legislation and institutional requirements. The patients/participants provided their written informed consent to participate in this study.

## Author contributions

TH, DH, and JM developed the study design. TH developed the research questions. TH and DH organized the data collection and preparation, and performed the statistical analyses. TH prepared the manuscript, supported by DH. All authors discussed the results and contributed to the final manuscript.

## Funding

We acknowledge the support of the German Research Foundation (Deutsche Forschungsgemeinschaft; DFG) and the Open Access Publishing Fund of the Osnabrück University to cover the open access publication fees.

## Conflict of interest

The authors declare that the research was conducted in the absence of any commercial or financial relationships that could be construed as a potential conflict of interest.

## Publisher’s note

All claims expressed in this article are solely those of the authors and do not necessarily represent those of their affiliated organizations, or those of the publisher, the editors and the reviewers. Any product that may be evaluated in this article, or claim that may be made by its manufacturer, is not guaranteed or endorsed by the publisher.

## Supplementary material

The supplementary material for this article can be found online at: https://www.frontiersin.org/articles/10.3389/fpsyg.2023.1099138/full#supplementary-material

## Footnotes

^1^From 1,406 participants who started the online survey, we excluded 812 participants without sufficient responses, 22 participants without sufficient German skills, 18 participants without telework experience, three participants who did not respond seriously, and three students as we aimed for a permanently employed working sample.

^2^As Golden (2021), Haun et al. (2022) and Troll et al. (2022)> were published after our data collection, we could not derive telework strategies from these sources.

^5^Greer and Payne (2014) also found telework strategies related to "use advanced technologies" (i.e., establishing a technological setup at home close to the on-site setup) being frequently mentioned by high performing teleworkers for which no separate subcategory emerged in the present study. However, we found the single related telework strategy "I use a setup that is technically close to the setup at my on-site workstation." to be positively associated with job performance in the present study matching Greer and Payne’s (2014) initial findings.

^6^However, whereas Troll et al. (2022) did not find goal-setting related self-control strategies (i.e., setting goals and deadlines, making to-do-lists) to be a unique predictor of job performance, goal-setting related telework strategies (e.g., setting goals, getting organized) were part of the subcategory conducive work attitude associated with job performance in the present study. A reason for the divergent findings might be controlling for other sets of self-control strategies in Troll et al. (2022). We also controlled for other subcategories of telework strategies in our supplemental analyses (see Supplementary Material Section B and Section C at https://osf.io/gqpdf/) and found the same pattern of results reported in the main paper. However, in the present study, goal-setting related telework strategies were integrated into the subcategory conducive work attitude. Thus, in the present study, goal-setting related telework strategies were not controlled for telework strategies related to conducive work attitude, whereas in Troll et al. (2022) goal-setting related self-control strategies were controlled for self-control strategies related to autonomous motivation. Indeed, Troll et al. (2022) found positive bivariate correlations between goal-setting related self-control strategies and job performance.

^7^The second set of self-control strategies identified as unique predictor of job performance in Troll et al. (2022) were strategies related to somatic condition (i.e., optimizing the physical state to work productively, for instance, through sufficient sleep, coffee consumption, wearing fresh clothes). Self-control strategies related to somatic condition were most similar to the present study’s telework strategies related to health-promoting measures (e.g., adapting work-day to bio-rhythm, eating healthy) for which we did not find a positive association with job performance. Future research might pick up on these divergent results and more closely examine impacts of telework strategies related to altering the somatic condition/health-promoting measures on job performance.
