# Supplementary material for: Associations between the implementation of telework strategies and job performance: Moderating influences of boundary management preferences and telework experience
Source: Front Psychol. 2023 Feb 16;14:1099138. doi: 10.3389/fpsyg.2023.1099138 (PMC9980715; doi:10.3389/fpsyg.2023.1099138)
Supplement: Supplementary file 1 [file Table_1.PDF]

Supplementary Material

Supplement A

TABLE A1 | Standard deviations of the implementation of telework strategies, bivariate correlations between telework strategies and job performance, and supplemental results to the multiple linear regressions predicting job performance in the main paper.

Encoding	SD_{Imp}	$r_{Imp, JP}$	p	β_0	p	β_{BMP}	p	β_{TE}	p	β_{Age}	p	β_{Gender}	p	β_{Space}	p	$\beta_{Children}$	p	R^2	$R^2_{Adj.}$	F	p
P_	1.62	.07		-0.03		0.00		**0.20**		0.02		0.01		-0.02		0.09					
P_PSWL	1.75	.06		-0.03		-0.01		**0.20**		0.02		0.01		-0.02		0.09					
P_PSWL_1_r	1.14	.02	.63	-0.05	.41	-0.01	.80	**0.23**	<.001	0.02	.72	0.01	.95	-0.02	.71	0.09	.42	**.06**	**.04**	**3.63**	<.001
P_PSWL_2	1.73	**.11**	.009	-0.01	.89	-0.02	.62	**0.19**	<.001	0.02	.68	0.01	.87	-0.02	.75	0.05	.66	**.06**	**.04**	**3.27**	<.001
P_PSWL_3_r	1.66	.06	.17	-0.03	.63	-0.01	.82	**0.22**	<.001	0.02	.72	-0.02	.85	-0.02	.67	0.12	.30	**.05**	**.04**	**3.16**	<.001
P_PSWL_4	1.63	**.13**	.002	-0.04	.50	-0.02	.73	**0.20**	<.001	0.00	.97	0.02	.84	-0.03	.61	0.13	.24	**.06**	**.05**	**3.76**	<.001
P_PSWL_5	1.71	.07	.093	-0.03	.63	-0.01	.83	**0.20**	<.001	0.02	.69	0.03	.75	-0.03	.52	0.10	.36	**.05**	**.04**	**3.15**	.001
P_PSWL_6	1.84	.07	.085	0.00	.95	0.00	.99	**0.19**	<.001	0.03	.60	0.00	.99	-0.03	.59	0.09	.44	**.06**	**.04**	**3.35**	<.001
P_PSWL_7	2.02	.05	.23	-0.03	.65	0.00	.92	**0.20**	<.001	0.02	.63	0.01	.92	-0.02	.67	0.09	.41	**.05**	**.03**	**2.70**	.004
P_PSWL_8	1.74	**.11**	.013	-0.01	.82	-0.03	.57	**0.20**	<.001	0.01	.91	0.01	.89	-0.03	.53	0.09	.41	**.06**	**.04**	**3.49**	<.001
P_PSWL_9	2.01	.02	.69	-0.02	.77	0.01	.90	**0.20**	<.001	0.03	.53	0.01	.95	-0.01	.82	0.08	.48	**.04**	**.03**	**2.61**	.006
P_PSWL_10	2.18	.04	.37	-0.03	.67	0.01	.90	**0.20**	<.001	0.02	.66	0.01	.91	-0.01	.79	0.09	.42	**.05**	**.03**	**2.68**	.005
P_PSWL_11	1.71	.05	.30	-0.01	.83	0.01	.89	**0.20**	<.001	0.01	.82	0.01	.94	-0.01	.81	0.09	.42	**.05**	**.03**	**2.91**	.002
P_PSWL_12	1.62	.04	.38	-0.04	.57	0.01	.88	**0.20**	<.001	0.02	.72	0.02	.85	-0.01	.84	0.10	.35	**.05**	**.04**	**3.08**	.001
P_CWE	1.48	.08		-0.03		0.00		**0.20**		0.02		0.01		-0.02		0.09					
P_CWE_1	0.95	**.11**	.01	-0.01	.89	0.00	.95	**0.19**	<.001	0.02	.75	0.01	.95	-0.01	.81	0.07	.51	**.06**	**.04**	**3.38**	<.001
P_CWE_2	0.94	**.15**	<.001	-0.02	.70	0.01	.77	**0.21**	<.001	0.00	.94	0.01	.87	-0.01	.89	0.10	.36	**.07**	**.06**	**4.37**	<.001
P_CWE_3	1.25	.04	.40	-0.02	.79	0.00	.96	**0.20**	<.001	0.03	.59	0.01	.93	-0.01	.81	0.10	.39	**.05**	**.03**	**2.78**	.003
P_CWE_4	1.46	.16	<.001	-0.03	.68	0.00	.97	**0.17**	<.001	0.00	.99	-0.02	.80	-0.03	.56	0.08	.46	**.07**	**.05**	**4.14**	<.001
P_CWE_5	1.42	**.17**	<.001	-0.04	.48	-0.01	.82	**0.17**	<.001	0.02	.74	0.01	.95	-0.04	.42	0.11	.32	**.08**	**.06**	**4.47**	<.001
P_CWE_6_r	1.85	.05	.23	-0.03	.59	0.01	.88	**0.22**	<.001	0.02	.74	0.03	.79	-0.01	.87	0.10	.38	**.06**	**.04**	**3.47**	<.001
P_CWE_7	1.79	.05	.23	-0.01	.83	0.01	.88	**0.20**	<.001	0.02	.65	0.00	.96	-0.02	.76	0.08	.47	**.05**	**.03**	**2.63**	.006
P_CWE_8	1.61	.05	.24	-0.05	.43	0.00	.98	**0.20**	<.001	0.02	.62	0.05	.63	-0.02	.71	0.11	.34	**.05**	**.03**	**2.93**	.002
P_CWE_9	1.64	.04	.33	-0.03	.68	0.00	.97	**0.20**	<.001	0.02	.65	0.02	.83	-0.02	.76	0.10	.39	**.05**	**.03**	**2.80**	.003
P_CWE_10	1.83	-.02	.62	-0.03	.59	0.00	.93	**0.20**	<.001	0.03	.53	0.02	.84	-0.02	.73	0.10	.38	**.05**	**.03**	**2.98**	.002
P_CWE_11	1.48	**.10**	.018	-0.04	.48	-0.02	.72	**0.20**	<.001	0.00	.98	0.04	.67	-0.01	.86	0.10	.37	**.06**	**.04**	**3.47**	<.001
T_	1.62	.03		-0.04		-0.01		**0.20**		**0.02**		0.02		-0.01		0.09					

Encoding	SD_{Imp}	$r_{Imp, JP}$	p	β_0	p	β_{BMP}	p	β_{TE}	p	β_{Age}	p	β_{Gender}	p	β_{Space}	p	$\beta_{Children}$	p	R^2	$R^2_{Adj.}$	F	p
T_TS	1.70	.04		-0.04		-0.02		**0.20**		0.02		0.02		-0.01		0.09					
T_TS_1	1.42	.03	.53	-0.02	.76	0.00	.99	**0.20**	<.001	0.02	.63	0.03	.75	-0.01	.84	0.08	.48	**.05**	**.04**	**3.21**	<.001
T_TS_2	1.38	**.17**	<.001	-0.01	.82	-0.04	.36	**0.20**	<.001	0.00	.94	0.02	.81	-0.01	.80	0.09	.42	**.08**	**.07**	**5.03**	<.001
T_TS_3	1.70	**.12**	.007	-0.04	.56	-0.02	.61	**0.18**	<.001	0.02	.71	0.03	.79	0.00	.96	0.07	.54	**.05**	**.03**	**2.76**	.004
T_TS_4	2.09	**.10**	.022	-0.07	.28	-0.03	.51	**0.20**	<.001	0.01	.89	0.05	.58	-0.01	.89	0.10	.38	**.07**	**.05**	**3.95**	<.001
T_TS_5	1.54	**.15**	<.001	-0.04	.55	-0.09	.064	**0.22**	<.001	-0.03	.53	0.01	.88	-0.01	.83	0.09	.38	**.10**	**.08**	**6.03**	<.001
T_TS_6	2.06	.03	.56	-0.03	.64	-0.01	.91	**0.19**	<.001	0.04	.41	0.02	.85	0.00	.94	0.08	.48	**.05**	**.03**	**2.68**	.005
T_TS_7	1.76	-.04	.39	-0.02	.75	0.02	.74	**0.20**	<.001	0.03	.52	0.02	.85	-0.01	.81	0.08	.45	**.05**	**.03**	**2.73**	.004
T_TS_8	1.78	.01	.78	-0.03	.62	0.00	.98	**0.21**	<.001	0.03	.60	0.02	.86	-0.03	.63	0.10	.35	**.06**	**.04**	**3.39**	<.001
T_TS_9	1.71	-.04	.41	-0.05	.41	-0.01	.82	**0.20**	<.001	0.03	.52	-0.01	.91	-0.03	.56	0.13	.24	**.07**	**.05**	**4.04**	<.001
T_TS_10	1.90	.02	.74	-0.05	.44	-0.01	.76	**0.19**	<.001	0.04	.47	0.04	.68	-0.02	.71	0.12	.30	**.04**	**.03**	**2.37**	.013
T_TS_11	1.62	-.02	.61	-0.05	.39	0.01	.91	**0.20**	<.001	0.03	.58	0.00	.97	-0.01	.81	0.09	.40	**.05**	**.04**	**3.05**	.001
T_TS_12	1.44	-.04	.41	-0.04	.56	0.01	.76	**0.21**	<.001	0.03	.56	0.02	.80	-0.03	.60	0.10	.37	**.05**	**.03**	**2.76**	.004
T_TF	1.50	.00		-0.04		0.01		**0.21**		0.01		0.01		-0.01		0.09					
T_TF_1	1.26	.07	.10	-0.03	.60	0.03	.52	**0.20**	<.001	0.02	.67	-0.01	.90	-0.01	.83	0.08	.49	**.06**	**.04**	**3.35**	<.001
T_TF_2	1.46	-.03	.57	-0.02	.72	0.02	.73	**0.20**	<.001	0.02	.74	0.01	.92	-0.02	.77	0.10	.39	**.05**	**.03**	**2.65**	.005
T_TF_3	1.67	-.04	.36	-0.04	.59	0.01	.87	**0.22**	<.001	0.00	.98	0.01	.95	0.00	.96	0.12	.31	**.06**	**.04**	**3.37**	<.001
T_TF_4	1.50	**.15**	<.001	-0.05	.41	0.04	.34	**0.20**	<.001	0.02	.64	0.04	.71	-0.02	.76	0.12	.29	**.08**	**.06**	**4.44**	<.001
T_TF_5	1.57	**-.13**	.004	-0.03	.62	-0.02	.60	**0.22**	<.001	-0.01	.90	0.03	.75	-0.01	.86	0.07	.53	**.06**	**.05**	**3.78**	<.001
T_TF_6	1.52	-.01	.89	-0.02	.81	0.02	.71	**0.23**	<.001	-0.01	.89	0.01	.90	0.00	.99	0.07	.53	**.06**	**.04**	**3.09**	.001
T_TF_7	1.52	-.02	.63	-0.07	.30	-0.02	.75	**0.21**	<.001	0.01	.88	0.00	.99	0.00	.95	0.11	.31	**.05**	**.04**	**3.15**	.001
B_	1.53	**.10**		-0.04		-0.01		**0.20**		0.02		0.03		-0.01		0.08					
B_BSWL	1.65	.06		-0.04		-0.02		**0.21**		0.01		0.02		-0.01		0.07					
B_BSWL_1	1.30	**.14**	.001	-0.02	.79	-0.03	.55	**0.20**	<.001	-0.01	.86	0.03	.74	0.00	.97	0.04	.69	**.07**	**.06**	**4.28**	<.001
B_BSWL_2	1.52	**.11**	.012	-0.05	.47	-0.04	.36	**0.20**	<.001	0.01	.85	0.03	.74	-0.01	.86	0.08	.49	**.06**	**.04**	**3.61**	<.001
B_BSWL_3	1.48	**.19**	<.001	-0.05	.37	-0.08	.078	**0.21**	<.001	-0.03	.48	0.11	.24	-0.03	.53	0.10	.36	**.13**	**.11**	**8.04**	<.001
B_BSWL_4	1.56	**.16**	<.001	-0.04	.54	-0.02	.61	**0.19**	<.001	-0.01	.89	0.07	.44	-0.02	.65	0.05	.63	**.07**	**.05**	**3.97**	<.001
B_BSWL_5	1.60	**.15**	<.001	-0.07	.29	-0.02	.58	**0.21**	<.001	-0.02	.64	0.06	.51	-0.04	.47	0.11	.30	**.08**	**.06**	**4.60**	<.001
B_BSWL_6	1.93	-.03	.53	-0.03	.60	0.01	.78	**0.20**	<.001	0.02	.66	0.00	.97	-0.01	.81	0.09	.40	**.05**	**.03**	**2.84**	.003
B_BSWL_7	1.90	-.02	.67	-0.04	.54	0.01	.85	**0.21**	<.001	0.02	.65	0.00	.96	0.00	.93	0.07	.54	**.06**	**.05**	**3.72**	<.001
B_BSWL_8	1.73	**.16**	<.001	-0.04	.52	-0.04	.41	**0.21**	<.001	0.01	.77	-0.02	.83	0.00	.95	0.03	.75	**.08**	**.06**	**4.50**	<.001
B_BSWL_9	1.76	**.11**	.012	-0.01	.82	-0.02	.64	**0.20**	<.001	0.03	.60	-0.01	.88	0.00	.99	0.05	.67	**.06**	**.04**	**3.11**	.001
B_BSWL_10	1.74	.01	.74	-0.04	.49	-0.01	.87	**0.21**	<.001	0.02	.66	-0.01	.93	-0.01	.86	0.08	.47	**.05**	**.04**	**3.13**	.001
B_BSWL_11	1.62	-.04	.40	-0.01	.91	0.01	.90	**0.19**	<.001	0.04	.45	0.00	.98	-0.01	.87	0.07	.54	**.05**	**.03**	**2.60**	.006
B_BSWL_12	1.92	.01	.90	-0.03	.60	0.00	.96	**0.22**	<.001	0.02	.69	0.00	.96	-0.02	.71	0.07	.52	**.06**	**.05**	**3.82**	<.001
B_BSWL_13	1.70	.03	.45	-0.04	.53	0.00	.93	**0.20**	<.001	0.03	.51	0.02	.85	-0.02	.72	0.07	.50	**.05**	**.03**	**2.98**	.002
B_BSWL_14	1.53	.01	.90	-0.03	.64	0.00	.96	**0.21**	<.001	0.01	.76	0.02	.85	-0.01	.87	0.08	.45	**.05**	**.03**	**2.84**	.003
B_BSWL_15	1.44	-.01	.74	-0.02	.69	0.01	.88	**0.21**	<.001	0.04	.45	0.02	.82	-0.02	.70	0.06	.60	**.05**	**.03**	**2.85**	.003
B_CWA	1.35	**.20**		-0.04		0.00		**0.18**		0.01		0.05		-0.02		0.08					

Encoding	SD_{Imp}	$r_{Imp, JP}$	*p*	β_0	*p*	β_{BMP}	*p*	β_{TE}	*p*	β_{Age}	*p*	β_{Gender}	*p*	β_{Space}	*p*	$\beta_{Children}$	*p*	R^2	$R^2_{Adj.}$	*F*	*p*
B_CWA_1	1.20	**.15**	<.001	-0.02	.79	0.00	.93	**0.18**	<.001	0.04	.42	0.02	.84	0.00	.97	0.07	.52	**.07**	**.05**	**3.92**	<.001
B_CWA_2	1.22	**.16**	<.001	-0.06	.37	0.02	.63	**0.19**	<.001	0.01	.84	0.07	.44	-0.01	.89	0.10	.37	**.07**	**.05**	**3.89**	<.001
B_CWA_3	1.15	**.44**	<.001	-0.08	.16	0.00	.93	**0.14**	<.001	-0.02	.65	0.16	.063	-0.06	.27	0.08	.42	**.22**	**.20**	**15.44**	<.001
B_CWA_4	1.21	**.26**	<.001	-0.03	.57	-0.02	.58	**0.18**	<.001	0.07	.16	0.06	.49	-0.01	.87	0.01	.93	**.12**	**.10**	**7.00**	<.001
B_CWA_5	1.13	**.31**	<.001	-0.06	.34	-0.03	.50	**0.17**	<.001	-0.06	.24	0.11	.24	-0.03	.52	0.09	.39	**.14**	**.12**	**9.05**	<.001
B_CWA_6	1.55	.06	.15	-0.02	.78	0.00	.95	**0.22**	<.001	0.02	.72	0.01	.93	-0.01	.91	0.08	.47	**.06**	**.04**	**3.22**	<.001
B_CWA_7	1.42	**.16**	<.001	-0.03	.57	0.01	.86	**0.19**	<.001	0.00	.97	0.03	.72	-0.02	.76	0.11	.32	**.08**	**.06**	**4.72**	<.001
B_CWA_8	1.61	.06	.15	-0.02	.71	0.01	.87	**0.19**	<.001	0.04	.40	0.00	.97	-0.02	.64	0.10	.36	**.05**	**.03**	**2.81**	.003
B_CWA_9	1.63	**.16**	<.001	-0.05	.44	0.02	.67	**0.19**	<.001	0.02	.69	-0.01	.89	-0.01	.77	0.09	.39	**.07**	**.06**	**4.20**	<.001
B_HPM	1.52	.02		-0.03		0.01		**0.20**		0.03		0.02		-0.01		0.09					
B_HPM_1	1.38	**.11**	.008	-0.04	.47	0.00	.96	**0.19**	<.001	0.03	.55	0.05	.61	-0.01	.80	0.12	.26	**.06**	**.04**	**3.57**	<.001
B_HPM_2	1.55	.05	.22	-0.03	.61	0.01	.87	**0.20**	<.001	0.03	.50	0.00	.99	-0.01	.89	0.09	.41	**.05**	**.03**	**2.78**	.004
B_HPM_3	1.55	.02	.58	-0.03	.68	0.00	.99	**0.20**	<.001	0.03	.50	0.00	.97	-0.01	.78	0.10	.39	**.05**	**.03**	**2.71**	.004
B_HPM_4	1.66	.00	.93	-0.03	.66	0.00	.92	**0.21**	<.001	0.03	.57	0.01	.91	-0.01	.80	0.07	.52	**.05**	**.04**	**3.16**	.001
B_HPM_5	1.43	**-.10**	.019	-0.04	.51	0.02	.72	**0.21**	<.001	0.03	.49	0.04	.70	-0.01	.83	0.07	.51	**.07**	**.05**	**3.93**	<.001
C_	1.54	**.13**		-0.01		-0.01		**0.19**		0.02		0.01		-0.02		0.07					
C_MA	1.66	**.09**		0.00		-0.02		**0.20**		0.02		-0.01		-0.03		0.06					
C_MA_1	1.47	**.17**	<.001	0.03	.59	-0.01	.83	**0.22**	<.001	-0.01	.78	-0.04	.64	-0.04	.48	0.02	.82	**.08**	**.06**	**4.14**	<.001
C_MA_2	1.69	.08	.064	-0.02	.80	-0.01	.76	**0.20**	<.001	0.03	.56	0.06	.51	-0.01	.89	0.05	.62	**.07**	**.05**	**3.94**	<.001
C_MA_3	1.66	.08	.10	0.00	.99	0.00	.93	**0.22**	<.001	0.02	.65	-0.01	.91	-0.05	.40	0.07	.52	**.06**	**.04**	**2.99**	.002
C_MA_4	1.81	.06	.19	-0.01	.92	-0.04	.45	**0.19**	<.001	0.02	.73	-0.04	.70	-0.01	.92	0.08	.48	**.05**	**.03**	**2.57**	.007
C_MA_5	1.68	**.16**	<.001	0.00	.94	-0.05	.29	**0.18**	<.001	0.02	.70	0.05	.64	-0.03	.66	0.06	.63	**.07**	**.05**	**3.39**	<.001
C_MA_6	1.62	.05	.32	0.03	.66	0.00	.93	**0.22**	<.001	0.02	.63	-0.07	.50	-0.05	.36	0.07	.51	**.06**	**.04**	**2.81**	.003
C_MA_7	1.68	.06	.23	-0.02	.79	-0.07	.16	**0.19**	<.001	0.02	.72	0.01	.96	-0.02	.75	0.10	.37	**.06**	**.03**	**2.54**	.007
C_KC	1.43	**.16**		-0.02		0.00		**0.19**		0.03		0.02		-0.02		0.08					
C_KC_1	0.92	**.22**	<.001	-0.01	.83	0.00	.95	**0.17**	<.001	0.01	.80	0.00	.96	-0.03	.60	0.08	.45	**.08**	**.06**	**4.61**	<.001
C_KC_2	0.98	**.21**	<.001	-0.04	.56	0.01	.85	**0.17**	<.001	-0.01	.87	0.03	.72	-0.02	.75	0.08	.44	**.08**	**.06**	**4.81**	<.001
C_KC_3	1.33	**.11**	.016	-0.02	.70	0.02	.63	**0.19**	<.001	0.04	.36	0.02	.87	-0.01	.83	0.07	.50	**.06**	**.04**	**3.42**	<.001
C_KC_4	1.49	**.21**	<.001	-0.03	.64	-0.02	.66	**0.19**	<.001	0.04	.42	0.04	.69	-0.02	.69	0.07	.54	**.09**	**.08**	**5.66**	<.001
C_KC_5	1.93	**.15**	<.001	-0.01	.89	0.01	.84	**0.19**	<.001	0.04	.46	0.01	.93	-0.02	.65	0.05	.64	**.08**	**.06**	**4.61**	<.001
C_KC_6	1.49	**.12**	.008	-0.05	.45	-0.02	.73	**0.21**	<.001	0.04	.41	0.03	.73	-0.01	.87	0.10	.37	**.07**	**.05**	**4.19**	<.001
C_KC_7	1.87	**.12**	.006	-0.02	.77	0.03	.55	**0.19**	<.001	0.03	.59	0.01	.91	-0.01	.88	0.08	.49	**.06**	**.04**	**3.39**	<.001

Note. Gender was dummy-coded (0/1 = female/male). Children was dummy-coded (0/1 = no/yes). Imp = implementation of telework strategy; JP = self-reported job performance; BMP = boundary management preferences; TE = telework experience; P_ = physical; T_ = temporal; B_ = behavioral; C_ = communicative; PSWL = physical separation of work and leisure; CWE = conducive work environment; TF = temporal flexibility; TS = temporal structure; BSWL = behavioral separation of work and leisure; CWA = conducive work attitude; HPM = health-promoting measures; MA = make arrangements; KC = keep connection; P_PSWL_1 = I regularly work outside my home.; P_PSWL_2 = I use technology facilitating to separate work

and leisure.; P_PSWL_3 = I occasionally change my workstation.; P_PSWL_4 = I exclusively work in a designated place.; P_PSWL_5 = I keep work materials in a separate, dedicated place.; P_PSWL_6 = I arrange my workstation visually like a typical office.; P_PSWL_7 = I physically separate my workstation from the rest of my living environment.; P_PSWL_8 = I do not conduct leisure activities at my workstation.; P_PSWL_9 = I use physical barriers as boundaries between work and leisure.; P_PSWL_10 = I use a separate, dedicated room for working.; P_PSWL_11 = I establish an atmosphere at my workstation that differs from the rest of my home.; P_PSWL_12 = I wear work clothes.; P_CWE_1 = I make sure there is sufficient light at my workstation.; P_CWE_2 = I wear comfortable clothes.; P_CWE_3 = I regularly air the room.; P_CWE_4 = I use a setup that is technically close to the setup at my on-site workstation.; P_CWE_5 = I set up a conducive work environment.; P_CWE_6 = I listen to music that helps me concentrate.; P_CWE_7 = I configure my workstation ergonomically.; P_CWE_8 = I set up a pleasant room climate.; P_CWE_9 = I reduce potential sources of distraction by placing them out of reach of my workstation.; P_CWE_10 = I personalize my workstation.; P_CWE_11 = I try to reduce distraction factors.; T_TS_1 = I have a set time routine to start the workday in the morning.; T_TS_2 = I structure my workday temporarily.; T_TS_3 = I schedule in advance when I will work in my home office and when I will work on-site.; T_TS_4 = I log my working hours.; T_TS_5 = I strictly separate my work time from my leisure time.; T_TS_6 = I have set days for working from home.; T_TS_7 = I take a regular lunch break at set times.; T_TS_8 = I have a set time routine for the transition from work to leisure.; T_TS_9 = I do not work beyond my working hours agreed with the employer.; T_TS_10 = I align my break schedule with the official break times of my organization.; T_TS_11 = I strictly adhere to set working hours.; T_TS_12 = I schedule regular breaks.; T_TF_1 = I am temporally flexible in handling urgent work requests.; T_TF_2 = I schedule my work time in order to get the most of my leisure time.; T_TF_3 = If my work is short on time. I'll “save it up” to make up for it in the next days.; T_TF_4 = I schedule my leisure time in order to get the most of my work time.; T_TF_5 = I flexibly transfer personal matters to times when I typically work.; T_TF_6 = If my leisure is short on time, I'll “save it up” to make up for it in the next days.; T_TF_7 = I flexibly transfer my work to times when I typically attend to personal matters.; B_BSWL_1 = I have a technological routine for the transition into work at the start of the work day.; B_BSWL_2 = I have a set technological routine facilitating the transition from work to leisure.; B_BSWL_3 = I maintain the same routines of my on-site work.; B_BSWL_4 = I avoid reading non-work related materials at work.; B_BSWL_5 = I attend to personal matters at work only when taking a break or during lunch hour.; B_BSWL_6 = I do not take work-related calls after hours.; B_BSWL_7 = I do not respond to work-related messages after hours.; B_BSWL_8 = I have a rule which leisure aspects are allowed to spill over into work and which not.; B_BSWL_9 = I have a rule which work aspects are allowed to spill over into leisure and which not.; B_BSWL_10 = I do not go back to work after hours.; B_BSWL_11 = I use breaks to strictly separate work time from leisure time.; B_BSWL_12 = I do not read work-related messages after hours.; B_BSWL_13 = I have set rituals facilitating the transition from work to leisure.; B_BSWL_14 = I avoid talking about work-related matters in leisure contexts.; B_BSWL_15 = I avoid talking about personal matters in work contexts.; B_CWA_1 = I value the benefits of working from home.; B_CWA_2 = I get organized at work.; B_CWA_3 = I show a particularly high level of dedication.; B_CWA_4 = I try to strengthen my supervisor's confidence in the quality of my work.; B_CWA_5 = I adjust my attitude and behavior to optimally focus and concentrate at work.; B_CWA_6 = I schedule tasks that can be done particularly well at home.; B_CWA_7 = I set personal daily goals at work.; B_CWA_8 = I take a short lunch break and quickly continue working to get done as much as possible.; B_CWA_9 = I practice self-praise.; B_HPM_1 = I pay attention to healthy eating.; B_HPM_2 = I adapt my work day to my bio-rhythm.; B_HPM_3 = I integrate exercise into my work day.; B_HPM_4 = I integrate outdoor activities into my work day.; B_HPM_5 = I regularly take a “power nap”.; C_MA_1 = I make arrangements with household members facilitating undisturbed work.; C_MA_2 = I make arrangements with

colleagues/supervisors/my employer about when I can(not) be reached regarding work.; C_MA_3 = I'll confront household members if agreements about work and leisure are violated.; C_MA_4 = I make arrangements with customers/clients about when I can(not) be reached regarding work.; C_MA_5 = I'll confront colleagues/supervisors/my employer if agreements about work and leisure are violated.; C_MA_6 = Household members make arrangements with me in order to limit my workload.; C_MA_7 = I'll confront clients/customers if agreements about work and leisure matters are violated.; C_KC_1 = I use various communication channels.; C_KC_2 = I keep connected via technology to respond to colleagues/supervisors/my employer/customers/clients quickly.; C_KC_3 = I make small talk with my colleagues/supervisors/employer.; C_KC_4 = I communicate expectations and progress with colleagues/supervisors/my employer.; C_KC_5 = I use modern communication technology with colleagues/supervisors/my employer such as instant messaging.; C_KC_6 = I seek social interaction after work.; C_KC_7 = I use technology to stay in personal contact with colleagues;
_r = recoded.
Results in bold are significant at the $p \leq .05$ level.

Supplement B

The pattern of results of the for each telework subcategory aggregated β-coefficients predicting job performance reported in the main paper remained robust when computing scales for telework strategy subcategories averaging the implementation of the respective composing telework strategies per participant and simultaneously entering these scales into a multiple linear regression predicting job performance (using *z*-scaled variables and including control variables), and thus, remained robust when controlling for the overlap among telework strategies from different subcategories (see Table B1). More specifically, the subcategories conducive work attitude (β = 0.34 [0.24, 0.43], $p < .001$) and keep connection (β = 0.21 [0.12, 0.30], $p < .001$) were the most important positive predictors of job performance. These were followed by conducive work environment (β = 0.04 [-0.07, 0.16], $p = .45$), temporal structure (β = 0.01 [-0.10, 0.13], $p = .81$), physical separation of work and leisure (β = 0.01 [-0.10, 0.13], $p = .81$), make arrangements (β = 0.00 [-0.10, 0.10], $p = .97$), behavioral separation of work and leisure (β = -0.03 [-0.16, 0.09], $p = .59$), temporal flexibility (β = -0.03 [-0.12, 0.05], $p = .44$), and health-promoting measures (β = -0.12 [-0.22, -0.03], $p = .011$). Thus, in accordance with the results reported in the main paper, telework strategies from the subcategories conducive work attitude and keep connection appeared to be the most important positive predictors of job performance, whereas boundary related telework strategies appeared to be less important. We decided to not base our main results on the multiple linear regression based on scales of telework strategy subcategories, as the Cronbach's alphas for the subcategories were in some instances quite low ($\alpha_{\text{Temporal flexibility}} = .56$) indicating that telework strategies within subcategories were still heterogeneous. Thus, we report the results in the main paper on the level of the individual telework strategies and descriptively summarize results for the subcategories making this heterogeneity transparent.

TABLE B1 | Multiple linear regression of telework strategy scales following the categorization in the main paper predicting job performance.

	β	95%-CI	*p*
Intercept	-0.09	[-0.20, 0.02]	.12
Age	0.00	[-0.09, 0.09]	.97
Gender	**0.18**	[0.01, 0.35]	.043
Space	-0.04	[-0.13, 0.06]	.46
Children	0.06	[-0.14, 0.27]	.54
Physical separation of work and leisure	0.01	[-0.10, 0.13]	.81
Conducive work environment	0.04	[-0.07, 0.16]	.45
Temporal structure	0.01	[-0.10, 0.13]	.81
Temporal flexibility	-0.03	[-0.12, 0.05]	.44
Behavioral separation of work and leisure	-0.03	[-0.16, 0.09]	.59
Conducive work attitude	**0.34**	[0.24, 0.43]	<.001
Health-promoting measures	**-0.12**	[-0.22, -0.03]	.011
Make arrangements	0.00	[-0.10, 0.10]	.97
Keep connection	**0.21**	[0.12, 0.30]	<.001
F (13, 495)	**8.72**		<.001
R^2	**.19**		
$R^2_{\text{Adj.}}$	**.16**		

Note. $n = 509$. Gender was dummy-coded (0/1 = female/male). Children was dummy-coded (0/1 = no/yes). CI = confidence interval.
Results in bold are significant at the $p \leq .05$ level.

Supplement C

The pattern of results of the for each telework subcategory aggregated β-coefficients predicting job performance reported in the main paper remained robust when simultaneously entering factor analytically identified scales averaging the implementation of the respective composing telework strategies per participant as predictors into a multiple linear regression predicting job performance (using *z*-scaled variables and including control variables), and thus, remained robust when controlling for the overlap among telework strategies from different factor analytically identified subcategories. To conduct this supplemental analysis, we first randomly split the data and computed an exploratory factor analysis (principal component analysis with varimax rotation computed with the psych package in R; Revelle, 2021; see Table C1) with one half of the data. Based on the scree-plot, we identified eight factors. We labeled these factors based on the rule that a factor received the same label as the majority of the composing telework strategies in the taxonomy of the main paper (three telework strategies were assigned to a different factor than proposed by the taxonomy of the main paper). The eight factors identified were physical separation of work and leisure (7 telework strategies, α = .89), temporal structure (4 telework strategies, α = .71), temporal flexibility (3 telework strategies, α = .53), behavioral separation of work and leisure (4 telework strategies, α = .90), conducive work attitude (3 telework strategies, α = .70), health-promoting measures (3 telework strategies, α = .66), make arrangements (5 telework strategies, α = .79), and keep connection (2 telework strategies, α = .65). Based on the other half of the data set, we computed a confirmatory factor analysis using maximum likelihood estimation (computed with the lavaan package in R; Rosseel, 2012; $\chi^2(406) = 614.68$, $p < .001$; CFI = .88; TLI = .87; SRMR = .075; RMSEA = .057). We then computed scales averaging the implementation of telework strategies per participant for each factor and entered these scales simultaneously in a multiple linear regression predicting job performance (see Table C2). The pattern of results was similar to the results of the for each telework strategy subcategory aggregated β-coefficients predicting job performance reported in the main paper. The factors conducive work attitude (β = 0.25 [0.16, 0.34], $p < .001$) and keep connection (β = 0.21 [0.13, 0.30], $p < .001$) were the most important positive predictors of job performance. These were followed by make arrangements (β = 0.05 [-0.04, 0.15], $p = .26$), physical separation of work and leisure (β = 0.04 [-0.05, 0.14], $p = .37$), behavioral separation of work and leisure (β = 0.02 [-0.07, 0.11], $p = .65$), health promoting measures (β = -0.04 [-0.13, 0.05], $p = .38$), temporal structure (β = -0.05 [-0.14, 0.04], $p = .30$), and temporal flexibility (β = -0.06 [-0.15, 0.03], $p = .18$). We decided to not base our main results on factor analytically identified telework strategy scales because the data generation process was not tailored to do so, leading to a somewhat arbitrary factor analytically identification of telework strategy categories (if previous research and popular media described similar telework strategies in detail factors would emerge, whereas if previous research and popular media described standalone, definite telework strategies no factors would emerge) and to a removal of a large proportion of telework strategies (potentially leading to overseeing relevant telework strategies for job performance). Considering that research on telework strategies is still in its’ infancy, the focus of the present study was to assess the broad range of heterogeneous telework strategies present in the scientific literature and popular media to paint a comprehensive, highly resolved picture.

TABLE C1 | Principal component analysis of the 85 telework strategies with varimax rotation.

Telework strategy	Factor loadings								Communalities
	PC2: P_PSWL	PC7: B_BSWL	PC1: T_TS	PC3: C_MA	PC4: B_CWA	PC6: B_HPM	PC5: C_KC	PC8: T_TF	
I regularly work outside my home. (recoded)		.27	.14	-.13			.13		.14
I use technology facilitating to separate work and leisure.	.28	.36	.21	.22			.24	.11	.37
I occasionally change my workstation. (recoded)	.28	.27	.32	-.11	-.11	-.20		-.16	.34
I exclusively work in a designated place.	.45	.18	.39	-.15					.43
I keep work materials in a separate, dedicated place.	.57	.16	.12		.29		.10		.46
I arrange my workstation visually like a typical office.	**.74**				.17	.18	.10		.63
I physically separate my workstation from the rest of my living environment.	**.86**								.74
I do not conduct leisure activities at my workstation.	.52	.23	.17		.18			-.20	.44
I use physical barriers as boundaries between work and leisure.	**.77**								.61
I use a separate, dedicated room for working.	**.81**								.68
I establish an atmosphere at my workstation that differs from the rest of my home.	**.66**		.15	.14	.11	.18			.52
I wear work clothes.	.29			.19	.15	.12		-.12	.18
I make sure there is sufficient light at my workstation.	.21	.16			.17	.48	.17		.36
I wear comfortable clothes.		.28		-.13	.14	.16	.15		.17
I regularly air the room.	.22				.12	**.62**			.47
I use a setup that is technically close to the setup at my on-site workstation.	.48		.13				.37		.39
I set up a conducive work environment.	**.62**				.22	.19	.19		.52
I listen to music that helps me concentrate. (recoded)				-.21	.16			-.20	.13
I configure my workstation ergonomically.	**.66**		.13			.25			.52
I set up a pleasant room climate.	.18			.19	.20	.37			.26
I reduce potential sources of distraction by placing them out of reach of my workstation.	.30			.16	.40	.19	-.18	-.14	.37
I personalize my workstation.	.28			.24		.11	.15		.20
I try to reduce distraction factors.	.31	.19	.12	.29	.34		-.19	-.26	.46
I have a set time routine to start the workday in the morning.	.13		**.65**		.14	.13	.12	-.17	.53

Telework strategy	Factor loadings								Communalities
	PC2: P_PSWL	PC7: B_BSWL	PC1: T_TS	PC3: C_MA	PC4: B_CWA	PC6: B_HPM	PC5: C_KC	PC8: T_TF	
I schedule in advance when I will work in my home office and when I will work on-site.			.42		.21			.32	.34
I structure my workday temporarily.	.21	.28	.36			.38	.16		.43
I log my working hours.		.45			.11		.27		.31
I strictly separate my work time from my leisure time.	.23	.52	.46	.14	.20	.12		-.14	.62
I have set days for working from home.	.20	.19	.40					.24	.30
I take a regular lunch break at set times.		.19	**.60**	.11	-.16	.26			.51
I have a set time routine for the transition from work to leisure.		.20	**.61**	.24	.11				.49
I do not work beyond my working hours agreed with the employer.	.11	.43	.35				-.10	.21	.38
I align my break schedule with the official break times of my organization.	.16	.24	.52			.15			.39
I strictly adhere to set working hours.		.31	**.64**	.15				-.12	.55
I schedule regular breaks.	.12		.17	.28		.43	-.14	.20	.38
I am temporally flexible in handling urgent work requests.	.23		-.48	-.15		.10			.35
I schedule my work time in order to get the most of my leisure time.						.11		**.65**	.45
If my work is short on time, I'll “save it up” to make up for it in the next days.	.12	-.14						**.51**	.30
I schedule my leisure time in order to get the most of my work time.	.16				.35	.14		.11	.19
I flexibly transfer personal matters to times when I typically work.		-.21	-.30		-.20			**.62**	.57
If my leisure is short on time, I'll “save it up” to make up for it in the next days.				.21				**.58**	.40
I flexibly transfer my work to times when I typically attend to personal matters.		-.58	-.32			.10		.20	.50
I have a technological routine for the transition into work at the start of the work day.	.18	.37	.21				.51		.49
I have a set technological routine facilitating the transition from work to leisure.	.13	.49	.25			.14	.40		.50
I maintain the same routines of my on-site work.	.19	.14	.52		.43		.13	-.14	.55
I avoid reading non-work related materials at work.	.27	.18	.15		.56			-.22	.50
I attend to personal matters at work only when taking a break or during lunch hour.	.16	.12	.37		.39	.13		-.24	.40
I do not take work-related calls after hours.		**.76**	.12	.22			-.10		.65
I do not respond to work-related messages after hours.		**.85**	.17	.13			-.10		.78
I have a rule which leisure aspects are allowed to spill over into work and which not.		.18	.30	.49	.16			-.11	.41

Telework strategy	Factor loadings								Communalities
	PC2: P_PSWL	PC7: B_BSWL	PC1: T_TS	PC3: C_MA	PC4: B_CWA	PC6: B_HPM	PC5: C_KC	PC8: T_TF	
I have a rule which work aspects are allowed to spill over into leisure and which not.		.28	.37	.47		.10			.44
I do not go back to work after hours.		**.77**	.12				-.11		.63
I use breaks to strictly separate work time from leisure time.	.15	.19	.41	.30		.42		.20	.53
I do not read work-related messages after hours.		**.83**	.19	.16					.75
I have set rituals facilitating the transition from work to leisure.		.19	.34	.32		.39			.42
I avoid talking about work-related matters in leisure contexts.	.23	.21	.28	.22	.10	-.10		.22	.29
I avoid talking about personal matters in work contexts.	.14	.25		.19	.19		-.19		.19
I value the benefits of working from home.		.22				.13	.33	.26	.25
I get organized at work.				.15	**.64**			.11	.46
I show a particularly high level of dedication.	.16				.57		.30		.47
I try to strengthen my supervisor's confidence in the quality of my work.				.11	.41		.34		.31
I adjust my attitude and behavior to optimally focus and concentrate at work.	.23	.11	.20		**.61**	.13			.50
I schedule tasks that can be done particularly well at home.			.23	.15	.45		-.13	.20	.35
I set personal daily goals at work.					**.64**	.18			.47
I take a short lunch break and quickly continue working to get done as much as possible.		-.21	-.20		.21	-.21	.21		.23
I practice self-praise.				.28	.11	.24	.34	.16	.30
I pay attention to healthy eating.			.20	.15	.26	.50			.39
I adapt my work day to my bio-rhythm.			-.14	.11	.15	.25		.52	.38
I integrate exercise into my work day.				.16	.19	**.60**			.43
I integrate outdoor activities into my work day.		-.11		.15		**.61**			.43
I regularly take a “power nap”.		-.19	.15	.10	-.13	.39		.27	.31
I make arrangements with household members facilitating undisturbed work.	.24	.15	.13	.44	.37				.44
I make arrangements with colleagues/ supervisors/my employer about when I can(not) be reached regarding work.		.20	.31	.49	.25		.10	.20	.50
I'll confront household members if agreements about work and leisure are violated.				**.68**	.12	.19			.53
I make arrangements with customers/clients about when I can(not) be reached regarding work.			.12	**.56**	.15		.14	.24	.44
I'll confront colleagues/supervisors/my employer if agreements about work and leisure are violated.		.21		**.77**		.16			.67

Telework strategy	Factor loadings								Communalities
	PC2: P_PSWL	PC7: B_BSWL	PC1: T_TS	PC3: C_MA	PC4: B_CWA	PC6: B_HPM	PC5: C_KC	PC8: T_TF	
Household members make arrangements with me in order to limit my workload.	.15	-.16		**.53**	.11	.17		.11	.39
I'll confront clients/customers if agreements about work and leisure matters are violated.		.16		**.79**		.18		.12	.69
I use various communication channels.							**.64**		.43
I keep connected via technology to respond to colleagues/supervisors/my employer/customers/clients quickly.					.21		**.61**		.42
I make small talk with my colleagues/supervisors/employer.			.11		-.27		.35		.22
I communicate expectations and progress with colleagues/supervisors/my employer.			.23	.37	.19		.36	.10	.37
I use modern communication technology with colleagues/supervisors/my employer such as instant messaging.		-.24		.21	-.14		.44		.33
I seek social interaction after work.	-.10	.14		.11		.38	.23		.26
I use technology to stay in personal contact with colleagues.		-.14	-.25	.24		.19	.46		.40
Eigenvalue	5.72	2.29	13.19	4.87	3.16	2.32	2.78	1.94	
Sum of squared loadings	6.31	5.73	5.28	4.77	4.29	3.75	3.19	2.96	
Proportion of variance (in %)	7.40%	6.70%	6.20%	5.60%	5.00%	4.40%	3.80%	3.50%	
Cumulative proportion of variance (in %)	7.40%	14.10%	20.30%	25.90%	30.90%	35.30%	39.20%	42.70%	

Note. *n* = 274. Factor loadings < |.10| are not displayed. Telework strategies in italics represent strategies being assigned to a different factor than in the categorization of the main paper. PC = principal component; P_ = physical; B_ = behavioral; T_ = temporal; C_ = communicative; PSWL = physical separation of work and leisure; BSWL = behavioral separation of work and leisure; TS = temporal structure; MA = make arrangements; CWA = conducive work attitude; HPM = health-promoting measures; KC = keep connection; TF = temporal flexibility.
Factor loadings >|.30| and loading >|.30| on the respective factor than on any other factor are bold.

TABLE C2 | Multiple linear regression of factor analytically identified telework strategy scales predicting job performance.

	β	95%-CI	*p*
Intercept	-0.07	[-0.19, 0.05]	.24
Age	-0.05	[-0.14, 0.05]	.33
Gender	0.12	[-0.06, 0.30]	.19
Space	-0.01	[-0.12, 0.10]	.85
Children	0.10	[-0.11, 0.31]	.35
Physical separation of work and leisure	0.04	[-0.05, 0.14]	.37
Temporal structure	-0.05	[-0.14, 0.04]	.30
Temporal flexibility	-0.06	[-0.15, 0.03]	.18
Behavioral separation of work and leisure	0.02	[-0.07, 0.11]	.65
Conducive work attitude	**0.25**	[0.16, 0.34]	<.001
Health-promoting measures	-0.04	[-0.13, 0.05]	.38
Make arrangements	0.05	[-0.04, 0.15]	.26
Keep connection	**0.21**	[0.13, 0.30]	<.001
F (12, 489)	**6.18**		<.001
R^2	**.13**		
$R^2_{Adj.}$	**.11**		

Note. n = 502. Gender was dummy-coded (0/1 = female/male). Children was dummy-coded (0/1 = no/yes). CI = confidence interval.
Results in bold are significant at the $p \leq .05$ level.

References

Revelle, W. (2021). *Psych: Procedures for psychological, psychometric, and personality research*. Northwestern University, Evanston, Illinois. R package version 2.1.9. https://CRAN.R-project.org/package=psych.

Rosseel, Y. (2012). Lavaan: An R package for structural equation modeling and more. Version 0.5–12 (BETA). *Journal of Statistical Software*, *48*(2), 1–36. https://www.jstatsoft.org/v48/i02/
